# Implicit Learning of True and False Belief Sequences

**DOI:** 10.3389/fpsyg.2021.643594

**Published:** 2021-03-26

**Authors:** Qianying Ma, Elien Heleven, Giulia Funghi, Min Pu, Kris Baetens, Natacha Deroost, Frank Van Overwalle

**Affiliations:** ^1^Department of Psychology and Center for Neuroscience, Vrije Universiteit Brussel, Brussels, Belgium; ^2^Department of Psychology, Sapienza University of Rome, Rome, Italy

**Keywords:** serial reaction time task, false belief task, social sequence learning, go/no go task, false photograph task

## Abstract

To investigate whether people can implicitly learn regularities in a social context, we developed a new implicit sequence learning task combining elements from classic false belief and serial reaction time tasks. Participants learned that protagonists were offered flowers at four locations. The protagonists' beliefs concerning the flowers were true or false, depending on their orientation, respectively, toward the scene (so that the flowers could be seen) or away from it. Unbeknown to the participants, there was a fixed belief-related sequence involving three dimensions (identity of the two protagonists, true-false belief orientation held by the protagonists, and flower location as believed by the protagonists). Participants had to indicate as fast as possible where the flowers were located (Experiment 1), or how many flowers were given (Experiment 2) according to the protagonists. Experiment 1 combined perceptual and motor processes (as both the belief-related sequence and motor responses referred to location), whereas Experiment 2 unconfounded the sequence and motor responses, allowing to investigate pure perceptual implicit learning. For reasons of comparison, two non-social conditions were created in Experiment 2 by replacing the protagonists with two non-social objects—colored cameras or shapes. Results revealed significant implicit sequence learning of all belief-related dimensions in Experiment 1, and of true-false belief orientation in Experiment 2, even without a motor confound. Importantly, there were faster reaction times and stronger sequence learning effects in the social than in the non-social conditions. The present findings demonstrate for the first time that people are able to implicitly learn belief-related sequences.

## Highlights

- This study explores implicit belief-related sequences learning in a social context.
- Participants implicitly learned a sequence of true-false belief orientations (without motor confounds).
- Implicit belief-related sequence learning was faster than structurally identical non-social learning.

## Introduction

In our daily life, we benefit from routine sequences of actions, even when we are relatively unaware of these regularities. For example, when we learn to ride a bike or drive a car, the crude time-course of simple initial movements evolves gradually into faster and fine-grained sequences, resulting in fluent and skilled movements. Quite often, these sequential actions are learned in an implicit manner, so that the resulting knowledge is hard to bring to consciousness. Based on this observation, we investigate here whether regularities in social environments are also learned in an implicit manner and so can facilitate the understanding of social behaviors and interactions with other people.

One critical condition for smooth social interactions is understanding the mental state of other people (e.g., beliefs, knowledge, traits etc.), also termed “mentalizing” or “Theory of Mind” (ToM; Premack and Woodruff, 1978; for reviews see Van Overwalle, 2009; Schurz et al., 2014; Molenberghs et al., 2016). This requires the understanding that people's social activities are often driven by what they believe and know. A key test to identify people's capacity to infer others' mental states is the false belief task (Wimmer and Perner, 1983; Rubio-Fernández, 2013; Kampis et al., 2017).

An example, which is similar to the present experiment, is the false belief task developed by Saxe et al. (2006). In this task, participants saw a girl and a chocolate being hidden in one of two boxes. The chocolate bar moved to the same or the other box when the girl was oriented toward or away from the boxes. The participants were asked to identify “where the girl thinks the chocolate bar is.” When the girl was oriented toward the boxes, the participants knew that she could see the movements of the chocolate bar and therefore held a true belief of reality. Conversely, when the girl was oriented away from the boxes, the participants had to realize that the girl could not see reality, and therefore held a false belief. They had to infer what the girl believed from the last time when she could observe the position of the chocolate. This required the participants to hold a representation in mind of the girl independently of their own belief.

Considering the dynamic nature of daily life, understanding a single false belief event is not enough. Quite often, social interactions require people to continuously monitor what other people see, know and believe, such as in joint actions, like playing football or basketball, or dancing with a partner (Kampis et al., 2017). Moreover, predictions of how other's mental states might change, may help people to anticipate what people's next beliefs are and how they may act, and prepare their reactions accordingly. More importantly, people often make such predictions about other's sequential mental states intuitively. Previous research investigated how social cues (i.e., eye gaze) modulate learning of motor sequences (Geiger et al., 2018). Although the results showed that social cues promote learning of sequences of actions, there is still a lack of research investigating to what extent people implicitly learn sequences that involve the attribution of beliefs, which are considered a fundamental capacity for social interaction.

In order to investigate whether people can implicitly learn sequences related to mental beliefs of others, we adjusted the classic serial reaction time (SRT; Nissen and Bullemer, 1987) task which measures implicit sequence learning. In a classic SRT paradigm, a target appears at one of four spatial locations and participants have to respond to each target's location by pressing one of four keys. However, unbeknownst to the participants, the target location follows a specific sequence (e.g., 4-1-2-3-1-4-2-3, with 1–4 corresponding to four locations on the screen). Although participants were never requested to learn anything, they showed a tendency to respond faster when the sequence was repeated over training (i.e., general learning effect). Importantly, learning of a specific sequence (i.e., sequence-specific learning) was revealed by slower reaction times when the learned sequence is interrupted by a random sequence, followed by a faster reaction time when the learned sequence is reintroduced (Nissen and Bullemer, 1987; Deroost and Coomans, 2018). Sequence learning in the SRT task is called implicit learning, because learning takes place without intention and little awareness, and the resulting knowledge is difficult to express verbally (Destrebecqz and Cleeremans, 2001).

**Figure 1:**
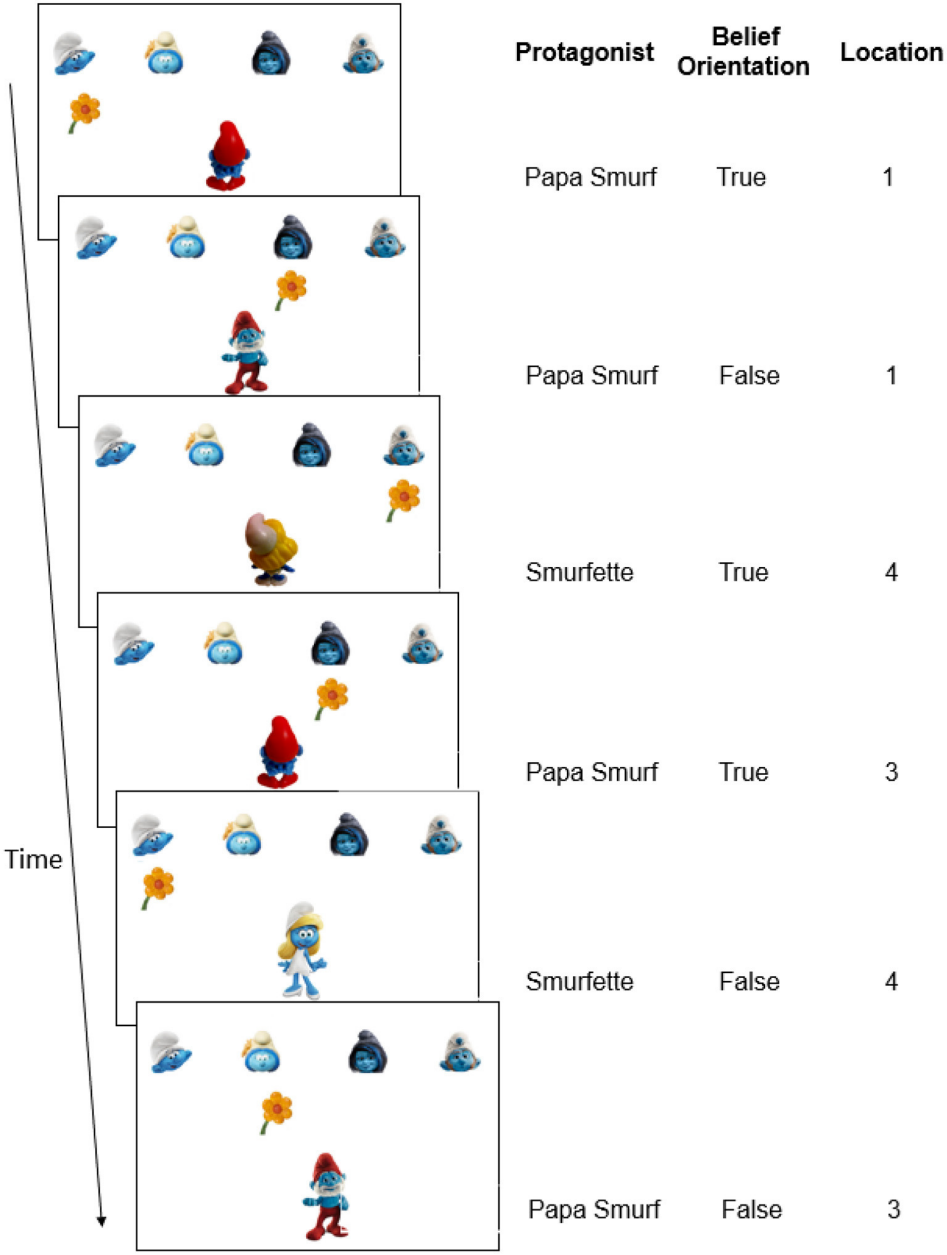
Schematic example of the belief SRT task in Experiment 1. **(Left)** On each trial, participants had to report where the flower was located from the protagonists' perspective (pressing “1”–“4” on the keyboard, corresponding to the leftmost to the rightmost location). When the protagonist was oriented toward the flowers (true belief), the correct answer is the flower's real location on the screen. When the protagonist was oriented away from the flowers (false belief), the correct answer is the location of the flower when the protagonist was previously oriented toward to the flowers. Consequently, in this example, the flower's location from the protagonists' perspective is 1-1-4-3-4-3 (i.e., the correct response). **(Right)** The belief-related sequence (first six trials only) with dimensions of the protagonists' identities (Papa Smurf or Smurfette), protagonists' belief orientations (true or false) and flowers' locations from the protagonist's perspective (1–4). In the full standard sequence which consisted of eight trials in Experiment 1, each location, protagonist identity and belief orientation was equally frequent (see **Table 1** and Supplementary Presentation 1). No other sequences were built in.

To investigate implicit learning of regularities in mental beliefs of others, we added false belief elements (Wimmer and Perner, 1983) to the classic SRT task (Nissen and Bullemer, 1987). In the current belief SRT task, participants saw two protagonists (i.e., Papa Smurf and Smurfette) receiving flowers from one of four other smurfs positioned at four fixed locations on top of the screen (Figure 1), and this was shown as a quick succession of the presentation of flowers and protagonists. Critically, as in the experiment by Saxe et al. (2006), participants were requested to take the perspective of the protagonist. Specifically, in Experiment 1, participants were asked to report the location of the flowers as the protagonist saw or knew it. When the protagonist was oriented toward the flowers (Figure 1, Trial 1), participants knew that he or she held a true belief about where the flowers were and who gave them. Conversely, when the protagonist was oriented away from the flowers (Figure 1, Trial 2), participants had to realize that the protagonist could not see any changes on screen and hence held an outdated and false belief about the flower's location. They were told that the protagonists still believed that everything was as before, so that the correct response on false trials was to repeat the response on the last true trial from the same protagonist. As in a classical SRT task, unbeknownst to participants, a sequence was embedded in the current task (Figure 1), in this case related to beliefs. This sequence involved three belief-related dimensions: the protagonist's identity (who holds the belief: Papa Smurf or Smurfette), the protagonist's true or false belief orientation (oriented toward or away from screen, respectively), and belief content (the location of the flowers as believed by the protagonists).

Three aspects of the current belief SRT task are critical. First, a false belief orientation involved a false belief in the sense that reality was unknown to the protagonist; which implied that participants did not need to compare the protagonist's belief with reality on the screen (otherwise it would have led to the inference that the belief was actually false or not). In fact, the present set-up was similar to the experiment by Saxe et al. (2006), mentioned earlier, where participants did not need to consider reality when the protagonist (the girl) was oriented away from the box. A false orientation in the present task reflects the most critical element of mentalizing, namely, participants had to realize that the protagonist did not know that an object could be changed, and therefore they had to infer what the protagonist believed independently from reality. This set-up was also similar to reduced-demand tasks for young children, where the critical object was removed from view so that children did not need to consider reality (see also Setoh et al., 2016; Grosso et al., 2019).

Second, there were two protagonists in the current task (Figure 1, Papa Smurf or Smurfette at the bottom). This was done to ensure that participants could attribute distinct mental states to each smurf, rather than simply remembering and repeating the last true trial. A necessary precondition for mentalizing is building an association between a protagonist and his/her own belief state (Bradford et al., 2015; Kampis et al., 2017). To build this association, detecting the potential protagonist can be considered a first step for mental state representations (Kampis et al., 2017). Having at least two protagonists who can hold a false belief encourages participants to detect and to continuously keep track of the perspective of each protagonist and to hold separate mental representations for each of them. In contrast, with a single protagonist who always appears on the screen, participants can provide correct responses without making any association with the protagonist, or even without taking the perspective of the protagonist. As noted earlier, this is not considered an authentic belief attribution (Bradford et al., 2015; Kampis et al., 2017).

Third, it is important to emphasize that the current study aimed to investigate implicit learning of belief-related sequences, not implicit understanding of the protagonists' true and false beliefs. In fact, the participants got on explicit instructions how to infer the protagonists' beliefs, and in particular, that the protagonists would hold false beliefs when they were oriented away from the flowers.

Similar to a classical SRT task, we hypothesize that participants can implicitly learn belief-related sequences (Coomans et al., 2011). In particular, we hypothesize (1) a general learning effect revealed by shorter reaction times for the repeated standard belief-related sequence, and additionally, (2) a sequence-specific learning revealed by longer reaction times when this sequence is disrupted. We also reasoned that at least the true-false belief orientation sequence will be learned implicitly, as this aspect of the task is critical for correct responding and it is also the key aspect of mentalizing.

## Experiment 1

In Experiment 1, we explore whether participants can implicitly learn a sequence involving multiple belief-related dimensions, and we also explore which dimensions they are able to learn.

### Method

#### Participants

Participants were 19 (Mean age = 20.42 ± 4.64 years, one male) students of the Vrije Universiteit Brussel who participated in the experiment in return for a course credit. Almost all participants were 1st year psychology students before they took any courses on cognitive psychology.

#### Apparatus and Stimuli

The experiment was run on personal computers with 17-inch screens and programmed in E-prime 2.0 (The program and related materials can be accessed *via* the Open Science Framework: https://osf.io/jhrpn/?view_only=12aeacb88fb048d18aa856f0603ed75b). Participants completed the task individually in cubicles of the psychology lab of the Vrije Universiteit Brussel. As shown in Figure 1, the target was a yellow flower, appearing on a white background in one of four horizontal locations. Four different little smurfs appeared on the top of the screen to mark the target flower's location. The two protagonists, Papa Smurf and Smurfette, were each shown individually at the bottom of the screen with their face orientated toward or away from flowers.

#### Procedure

Participants were told that “Papa Smurf or Smurfette (at the bottom of the screen) sees flowers, given by one of four smurfs at the top of the screen. One of the four smurfs will give a flower while Papa Smurf or Smurfette is watching (facing the screen) or not watching (facing you). You must indicate the smurf that gave the flowers (1°, 2°, 3°, or 4°) as seen by Papa Smurf or Smurfette.” It was explained that the four smurfs were numbered by their position on the screen, 1–4, from left to right. Next, an explicit mentalizing instruction was given: “throughout the task, you have to follow from whom Papa Smurf or Smurfette think they get a flower (which of the four smurfs at the top of the screen)” and “If they turned their back to the four smurfs, you have to indicate from whom Papa Smurf or Smurfette remember they received a flower the last time” (translated from Dutch). Participants were instructed to respond as fast as possible. They then went through a written example and were informed that they could ask questions if they failed to understand the instructions. During the experiment, they also went through a number of practice trials, as detailed below.

Each trial started after a response-stimulus interval of 400 ms as in previous research (Coomans et al., 2011). When participants made a wrong response or when they did not respond within 3 s, the word “Error” appeared (“Fout” in Dutch) for 750 ms on the screen. After each block, participants received feedback about their mean reaction time and error rate, and they were encouraged to make <5% errors. Participants got a break of 15 s after every two blocks.

A Practice Phase of two blocks of 24 trials preceded the main experiment, using a sequence which was different from the main experiment.

**Figure 2:**
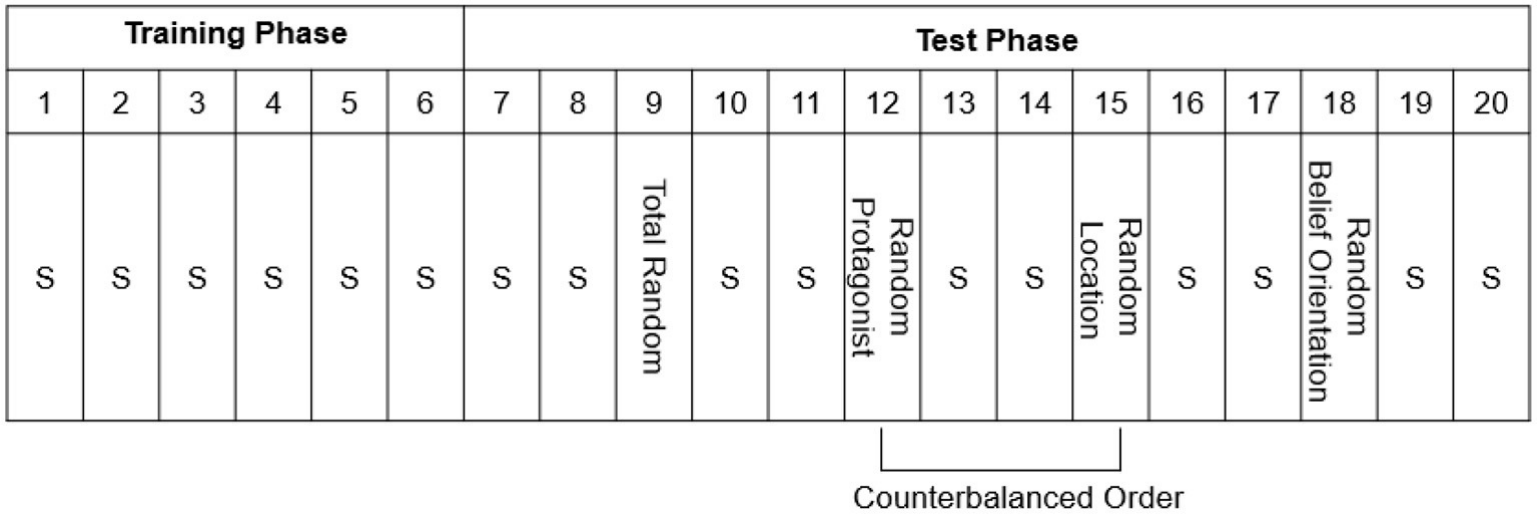
The procedure of Experiment 1, with Blocks numbered 1–20. S = Standard Blocks (with fixed belief-related sequence). Note that every block has a length of 48 trials. One random sequence was created for all dimensions together (i.e., Total Random), and three new sequences were created to test implicit learning of each belief-related dimension (i.e., Random Protagonist, Random Belief Orientation, and Random Location). After the main experiment shown here, there was a recognition test and a post-check question to measure awareness of the embedded sequence.

Afterwards, the participants completed the Training Phase (Figure 2), consisting of Standard Blocks 1–6 of 48 trials each. Unbeknownst to the participants, a fixed belief-related sequence consisting of eight trials (which was continuously repeated) was embedded in each block (Table 1). The Test Phase (Blocks 7–20) consisted of 14 blocks of 48 trials each, and consisted of 10 Standard Blocks, identical to those in the Training Phase, and four Random Blocks in which the Standard sequences was altered (Figure 2). The Random Blocks were created by altering one belief-related dimension whereas the other dimensions remained identical to the Standard Sequence: the sequence of the protagonists' identity was replaced by a new sequence (Random Protagonist), and likewise for the protagonists' belief orientation (Random Belief Orientation), and the flower's location (Random Location). One additional random block was created for all dimensions together (Total Random) in which all the dimensions (protagonists' identity, protagonists' belief orientation, and flower's location) were programmatically determined at random at each trial, with the restriction that the sequence always started with a true trial for each protagonist, and that no sequence of the same belief dimension occurred on more than two consecutive trials. The Total Random block was always provided in Block 9; for halve of the participants the Random Protagonist block was provided in Block 12 while the Random Location block was provided in Block 15, whereas the other half of participants received the reverse ordering of Blocks, and the Random Belief Orientation was always provide in Block 18. The Random Belief Orientation block was kept last, because we surmised that belief orientation was the most conspicuous sequence and hence would be learned easiest, so that this implicit knowledge would survive the testing of the other Random Blocks (Supplementary Table 1).

**Table 1:** Standard sequence embedded in the Training and Test Phase.

**Experiment 1: Standard Sequence to be learned (repeated every 8 trials)**																
Location (as believed by protagonists)	1	1	4	3	4	3	2	2								
Protagonist	M	M	Fe	M	Fe	M	Fe	Fe								
Belief orientation	T	Fa	T	T	Fa	Fa	T	Fa								
Prior true trial		−1			−2	−2		−1								
**Experiment 2: Standard Sequence to be learned (repeated every 16 trials)**																
Location (as believed by protagonists)	1	1	4	3	4	3	2	2	2	1	1	4	3	4	3	2
Protagonist	M	M	Fe	M	Fe	M	Fe	Fe	Fe	Fe	Fe	M	Fe	M	Fe	M
Belief orientation	T	Fa	T	T	Fa	Fa	T	Fa	Fa	T	Fa	T	T	Fa	Fa	T
Prior true trial		−1			−2	−2		−1	−2		−1			−2	−2	

*M, male; Fe, female; T, true; Fa, false. In Experiment 1, the correct response is the location as believed by the protagonists; in Experiment 2, the correct response is the number of flowers, which is determined randomly for each trial*.

#### Assessment of Awareness

Immediately after the main experiment, in order to assess participants' explicit awareness of the standard sequence, participants took a recognition test. On each trial, participants saw the same four smurfs at the top of the screen, and a transition of flowers from one location (i.e., smurf) to another, without any other information (i.e., protagonists) on the screen. They were asked to report whether this transition had been seen by Papa smurf or Smurfette in the main experiment (Yes or No). There were 16 trials involving all possible transitions, presented in a random order.

Afterwards, participants filled a post-check question in which they were asked “Did you notice anything particular during the experiment?”

#### Statistical Analysis

First, a general learning effect during the Training Phase was tested by a repeated measure analysis of variance (ANOVA) with Standard Blocks (1–6) as within-participant factor. Second, given our cautious hypothesis with respect to the belief-related dimensions (i.e., at minimum an effect for the true-false belief orientation dimension), a sequence-specific learning effect during the Test Phase for each belief-related dimension (and for all dimensions together, i.e., Total Random) was tested by separate paired *t*-tests between Random Blocks and Standard Blocks (the two adjacent Standard Blocks, one before and one after the Random Blocks, were collapsed in this analysis). The significance level was set to 0.05, and two-tailed tests were applied. When the sphericity assumption was violated for ANOVA, the Greenhouse-Geisser correction is reported. Finally, explicit awareness of the standard sequence was investigated, to ensure that learning was implicit.

To identify the minimum size of the effect that can be reliably detected, we applied a sensitivity power analysis on our experiment using G^*^power 3.1.9.4 (Faul et al., 2009). For the repeated ANOVA during the Training Phase, the alpha significance criterion was set to 0.05, the standard power criterion was set to 80%, the number of groups was 1, and the number of repeated measurements was 6 (Standard Blocks 1–6). This analysis indicated that the minimum effect size η^2^ should be 0.06. The sensitivity analysis for paired *t*-test during the Test Phase with the same significant level indicated that the minimum effect size of *Cohen's d* should be 0.70. As shown in the result section, the significant results in the Experiment 1 met these effect sizes' requirements.

A Bayes factor analysis was used to evaluate any effects found in the experiment (JASP0.14.1.0). Here, we relied on Bayes factors (BF_10_) for interpreting our main results. BF_10_ indicates the Bayes factor in favor of alternative hypothesis (H_1_) over null hypothesis (H_0_). For example, if BF_10_ = x, this means that the data are x times more likely under the alternative hypothesis (e.g., there are general and sequence-specific learning effects) than under the null hypothesis (e.g., there is no learning effect). BF_10_ >3 indicates substantial evidence to support the alternative hypothesis and BF_10_ >10 indicates strong evidence to support the alternative hypothesis (van Doorn et al., 2020).

### Results

Participants' error rates and mean reaction times (RTs) were analyzed. For the RTs analysis, responses during and immediately after an error were excluded. The results of one participant were omitted from the analyses because of an excessive error rate (24%), which was identified as an outlier (i.e., above the 3rd quartile plus 1.5 times the interquartile range, i.e., 1st vs. 3rd quartile difference). The mean error rate for the remaining participants was 5%, *SD* = 3%. Our hypotheses were supported by the following statistical analyses.

#### General Learning Effect During Training Phase

**Figure 3:**
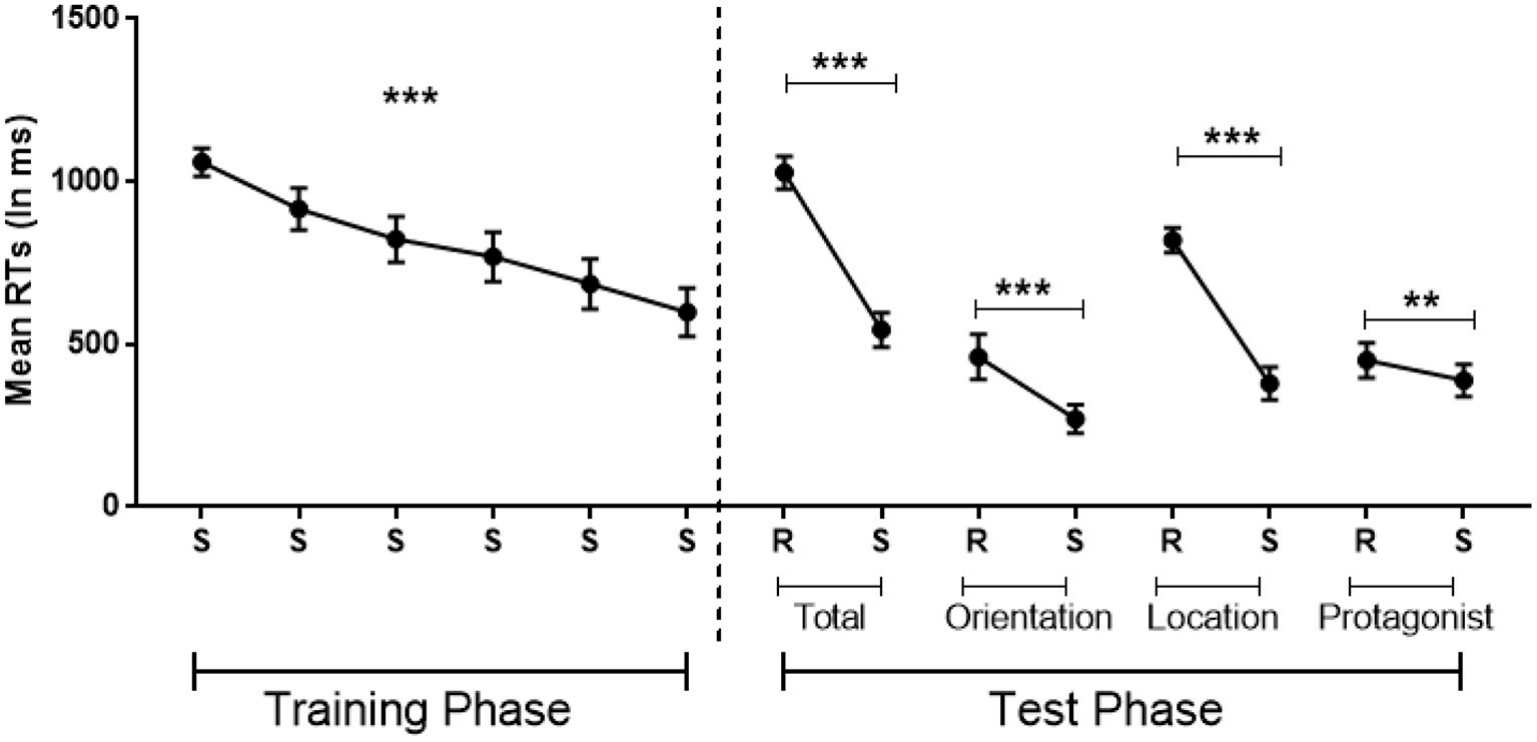
Implicit learning of the belief-related sequence in which the sequence is standard (S) or random (R), with randomization involving belief orientation (Orientation: true or false), the flower's location (Location: 1–4) or the protagonists identity (Protagonist: Papa smurf or Smurfette) or these three dimensions together (Total). Error bars = Standard errors of the mean. Standard blocks adjacent Random blocks of the same dimension were collapsed in the analysis. **p < 0.01; ***p < 0.001.

A repeated ANOVA with Standard Blocks (1–6) as within-participant factor showed implicit learning as attested by a significant decrease in error rates [*F*_(2.63,44.74)_ = 4, *MSE* = 13*, p* = 0.02 η^2^ = 0.19, BF_10_ = 13] and RTs [*F*_(5,85)_ = 25.28, *MSE* = 945,708, *p* < 0.001, η^2^ = 0.60, BF_10_ = 2.973e+12; see Figure 3 left].

#### Sequence-Specific Learning During Test Phase

Paired *t*-tests revealed that, compared with the adjacent Standard Blocks, error rates were significantly larger in the Total Random Block (Total Random: *M* = 9%, *SD* = 5%; Standard: *M* = 4%, *SD* = 3%; *t* = 3.28, *p* < 0.01, *Cohen's d* = 0.77, BF_10_ = 10) and the Random Location Block (Random Location: *M* = 9%, *SD* = 7%; Standard: *M* = 2%, *SD* = 2%; *t* = 3.93, *p* < 0.01, *Cohen's d* = 0.93, BF_10_ = 35), but failed to reach significance in the other two Random Blocks (all *p* > 0.5).

Moreover, RTs were significantly slower in all four Random Blocks compared with the Standard Blocks, demonstrating a significant effect of sequence-specific learning in all four Random Blocks (*t*_Total Random−*Standard*_ = 8.22, *p* < 0.001, *Cohen's d* = 1.93, BF_10_ = 62,523; *t*_Random Belief Orientation−*Standard*_ = 4.40, *p* < 0.001, *Cohen's d* = 1.04, BF_10_ = 85; *t*_Random Location−*Standard*_ = 8.52, *p* < 0.001, *Cohen's d* = 2.02, BF_10_ = 108,433; *t*_Random Protagonists−*Standard*_ = 2.77, *p* = 0.013, *Cohen's d* = 0.65, BF_10_ = 4; Figure 3 right). These tests survive a Bonferroni correction, with Random Protagonist just falling above threshold (at *p* = 0.052), although this correction is too stringent since the three dimensions are not totally independent.

#### Explicit Awareness

The recognition test immediately after the main experiment, yielded an average accuracy of 47% which is at chance level (50%).

In the post-check question, where participants were asked “Did you notice anything particular during the experiment?”, 11 out of 18 answered affirmatively. Of these 11 participants, 10 reported they noticed a pattern, and one left the answer blank. All 11 participants were treated as the “aware” group, no matter what they were aware of, because any kind of awareness may lead to potential sequence knowledge. For these participants, the average accuracy rate on the recognition test was 49%. The other participants were treated as the “unaware” group. To investigate whether the sequence learning effect was attributable to explicit awareness, we reran the previous RT analyses for general and specific implicit learning, now using mixed-ANOVAs with Awareness (aware vs. unaware) as an additional between-participant factor, to check any modulation by this factor. However, there was only a main trend of Awareness on general learning (*p* = 0.07, BF_10_ = 1) and no main effect on sequence-specific learning effects (all *p*s > 0.10), indicating that awareness tends at most to lead to numerically faster responses. More importantly, there was no interaction with any Random vs. Standard Block (all ps > 0.50, for details, see Supplementary Materials), indicating that any potential awareness did not lead to stronger learning effects on the belief-related sequences.

### Discussion

Experiment 1 confirms that participants could continuously infer protagonists' beliefs, as attested by the high rate of correct responses. More importantly, although participants were not requested to learn anything, the results provide evidence that they learned the embedded belief-related sequence, shown by the general learning and sequence-specific learning effects, during the Training and Testing Phases, respectively. The results further provided evidence of implicit learning for three belief-related dimensions (i.e., protagonist, belief orientation, and flower's location from protagonists' perspective). This suggests that people can implicitly learn who they will interact with during a series of social encounters, and whether the other person has true or false beliefs during that interaction.

A limitation of this experiment is that the sequence of the flowers' location on screen was confounded with the sequence of participants' responses. That is, perceptual information about the flower's location believed by protagonists lead to the same motor responses, so that participants may have learned the sequence of flower locations because they were aided by the implicit learning of the parallel motor sequences (Deroost and Soetens, 2006). This could explain in part the stronger specific learning effect (i.e., largest RT difference between Random and Standard Blocks) of the Location dimension in comparison with the other two belief-related dimensions (i.e., Belief Orientation and Protagonists, Figure 3). Consequently, it is possible that participants only learned the motor sequence rather than the belief-related sequence. However, this possibility provides no convincing explanation for the specific learning effects of the Belief Orientation and the Protagonists dimensions as motor responses were unrelated to these sequences. Nonetheless, it is still possible that the facilitation by concurrent motor learning of the Location sequence provided more mental resources to implicitly learn the Belief Orientation and Protagonists dimensions.

Some participants reported potential explicit awareness about the sequences. However, self-reported awareness did not have significant effects on sequence learning. It most likely reflects a “feeling of familiarity” rather than explicit sequence knowledge (Werheid et al., 2003). However, the fact that there were a limited number of participants in the aware and unaware group, prevents us from drawing any firm conclusions. Therefore, this finding needs to be confirmed in future research with more sensitive measures of explicit awareness.

## Experiment 2

Although Experiment 1 demonstrated that participants can learn belief-related sequences, several issues remained unanswered. As noted earlier, one limitation was that the sequence of the target location believed by the protagonists was confounded with the sequence of participants' responses. This is a typical confound in a classical SRT design (Nissen and Bullemer, 1987). However, in daily life, our interpretation of others' mental states does not always depend on our immediate motoric reactions to them. For example, people can understand a false belief task as an observer without really interacting with the protagonists (Saxe et al., 2006). Also, the parallel motor sequence may have contributes to a higher awareness of the sequence.

**Figure 4:**
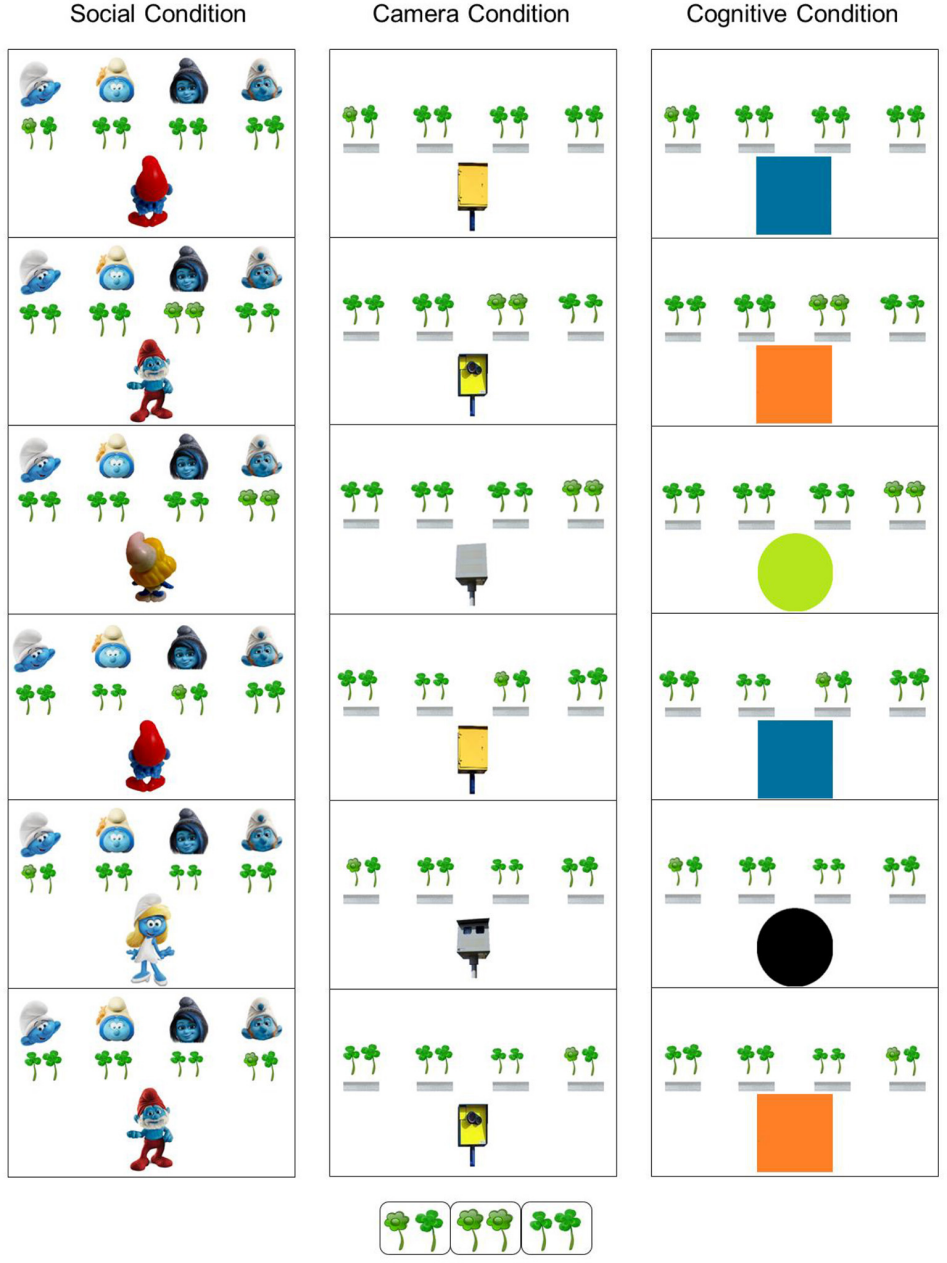
Schematic example of the belief SRT task in Experiment 2, showing the first six trials of the embedded sequence. On each trial, participants had to report the amount of green flowers (pressing “1” or “2” on the keyboard), as seen by the smurf (**Social Condition**), on the photos (**Camera Condition**), or depending on a shape's color (**Cognitive Condition**). In all conditions, the amount of flowers was determined by the orientation of the smurf or camera (toward vs. away from the flowers) or the color of the shape (green/blue vs. orange/black). Unknown to the participants, there was a fixed 16-trial sequence of the Protagonist (smurf/camera/shape), Orientation (belief orientation/camera orientation/color of shapes) and flowers' Location (for the full sequence, see Table 1 and Supplementary Presentation 2). The amount of flowers was completely random, hence making the response unpredictable from trial to trial, and dissociating sequence learning from motor responses. **[Inset Bottom**] The inset shows a display in which one flower is surrounded by one clover (as a distraction; of approximately the same shape and color), a display with two flowers, and a display with two clovers.

Hence, in Experiment 2, we tested whether participants could implicitly learn a belief-related sequence without motor confound, by dissociating the correlation between the sequence and motor response. We did so by asking participants to indicate the number of flowers offered to a smurf rather than their location; the number of flowers for every trial was random (Figure 4). Consequently, all three sequence dimensions (i.e., Location, Belief Orientation, and Protagonist) are unrelated to the correct response.

Given that the flower's location was now unrelated to responding, participants might largely ignore this sequence dimension. Hence, we attempted to make this aspect of the task more sensitive by flanking the flowers by distractors (i.e., clovers) so that each location showed two targets (flowers and/or clovers; Figure 4). Coomans et al. (2011) showed that increased perceptual demands (e.g., flankers that share perceptual features with the target) facilitates the expression of learned perceptual sequences of locations. This is because participants benefit little from learning to anticipate the location when they are able to immediately spot the flowers as in Experiment 1 (i.e., because they have a distinct, orange, color, and because they stand out alone against a white background). Conversely, when participants have to search for the target flower between flankers of the same color as in Experiment 2, they still might benefit by learning to anticipate the correct location of the target flower. Consequently, this set-up might promote learning of location in Experiment 2.

Although Experiment 1 demonstrated that implicit sequence learning is possible in a social context, a limitation of this experiment was that it is not entirely clear to what extent the results are specific to a social context or are determined by general processes that exist also outside this context. To investigate this, we added two non-social Conditions that are structurally identical to the Social Condition, but without any social elements. This was done by replacing the two protagonists by two non-social objects.

A first non-social condition is a Camera Condition, which requires participants to infer whether a photo taken by a camera was consistent with current reality or outdated. This condition is also known as the false photograph task, often used in false belief research (Saxe, 2006; Apperly et al., 2007). In particular, Papa Smurf and Smurfette were replaced by yellow and gray cameras, and belief orientation was replaced by the cameras' orientation (i.e., cameras' lens orientated toward or away from the flowers, Figure 4). Specifically, a “true belief” trial was represented by a camera which was orientated toward the flowers, indicating that the camera could take a “current photo” of the flowers, while a “false belief” trial was represented by a camera which was orientated away from the flowers, indicating that the camera only kept an “outdated photo.”

A second non-social condition is a Cognitive Condition (Figure 4). In particular, Papa Smurf and Smurfette were replaced by colored squares and circles, and belief orientation was replaced by colors with explicit instructions on how to use the information on the screen. Specifically, a “true belief” was represented by a blue square or a green circle indicating that information on flowers had to be taken from the current screen, while a “false belief” was represented by an orange square or a black circle indicated that information had to be taken from the previous trial from the same object. Thus, the four distinct pictures used in the Social Condition (i.e., to present the two smurfs in two distinct orientations), were replaced by four distinct pictures of colored shapes in the Cognitive Condition. The latter condition is similar to a Go/No-Go task (Rothmayr et al., 2011), where “Go” and “No-go” reflect “true” and “false” from the Social Condition, respectively.

To summarize, the Social Condition in Experiment 2 is in many respects similar to Experiment 1, except that the motor responses are now dissociated from the belief-related sequence, and clovers now flank the target flowers. Moreover, we added two non-social Camera and Cognitive Conditions, which share an identical structure and logic as the Social Condition, but exclude any social element.

We put forward the same hypotheses as Experiment 1 for the general learning and specific-sequence learning effects. In addition, as previous studies showed faster RTs in belief reasoning as opposed to non-social reasoning (Saxe et al., 2006; Cohen and German, 2010; Callejas et al., 2011), we hypothesized faster responses of the belief-related sequences in the Social Condition as opposed to the non-social Conditions. We also reasoned that, if confirmed, this may leave more mental resources available to detect sequential knowledge in the context of the Social Condition in comparison with the other non-social Conditions.

### Method

#### Participants

For the Social Condition, participants were 42 students (Mean age = 19.88 ± 1.98 years, 9 males); for the Camera Condition, participants were 43 students (19.86 ± 2.86 years, 11 males), and for the Cognitive Condition, participants were 41 students (19.54 ± 3.31 years, 3 males) of the Vrije Universiteit Brussel who participated in return for a course credit and were drawn from the same population as Experiment 1. We doubled the sample size of the previous Experiment 1 here because perceptual sequence learning is vulnerable to stimulus complexity and difficult to demonstrate compared to motor learning (Kelly and Burton, 2001; Coomans et al., 2011), and because no earlier studies investigated perceptual sequence learning with multiple belief-related dimensions. In this way, we could ensure that the lack of any significant differences in learning was not due to the limited sample size.

#### Apparatus, Stimuli, and Procedure

Apparatus, stimuli, and procedure were the same as in Experiment 1, except for the following changes described below.

In the Social Condition, the target consisted of one or two green flowers, flanked by green clovers as distractors (Figure 4) described as “of no importance.” The number of flowers was randomly determined at every trial. Participants were introduced to the task in the same manner as in Experiment 1. However, for this experiment they were instructed to “indicate how many flowers are given (1 or 2), as seen by Papa Smurf or Smurfette.” It was further detailed that “throughout the task you have to follow how many flowers Papa Smurf or Smurfette think they receive. If they are turned with their back to the four smurfs, you have to indicate how many flowers they (remember that they) received the last time” (translated from Dutch). They went through an example, and were informed that they could ask questions if they failed to understand the instructions.

In the Camera Condition, stimuli were the same as in the Social Condition, except for the following changes (Figure 4). Four small pictures of the curb of a footpath appeared on the top of the screen (instead of the four little smurfs), and the flowers and clovers appeared on top of them. Two distinct fully automated traffic cameras in a yellow or gray color were displayed at the bottom of the screen (instead of Papa Smurf or Smurfette). Each of these cameras could be orientated either toward or away from the screen. Participants were instructed: “When the camera is facing the flowers, you should indicate the number of flowers in the current photo. When the camera is facing you, indicate the number of flowers in the previous photo when that same camera was facing the flowers” (translated from Dutch).

In the Cognitive Condition, stimuli were the same as in the Camera Condition, except for the following changes (Figure 4). A colored square or circle appeared at the bottom of the screen (instead of cameras). Participants were instructed: “When a blue square or a green circle appears, you should indicate how many flowers there are (1 or 2). When the square is orange, repeat the previous number of flowers from the blue square. When the circle is black, repeat the previous number of flowers from the green circle” (translated from Dutch).

**Figure 5:**
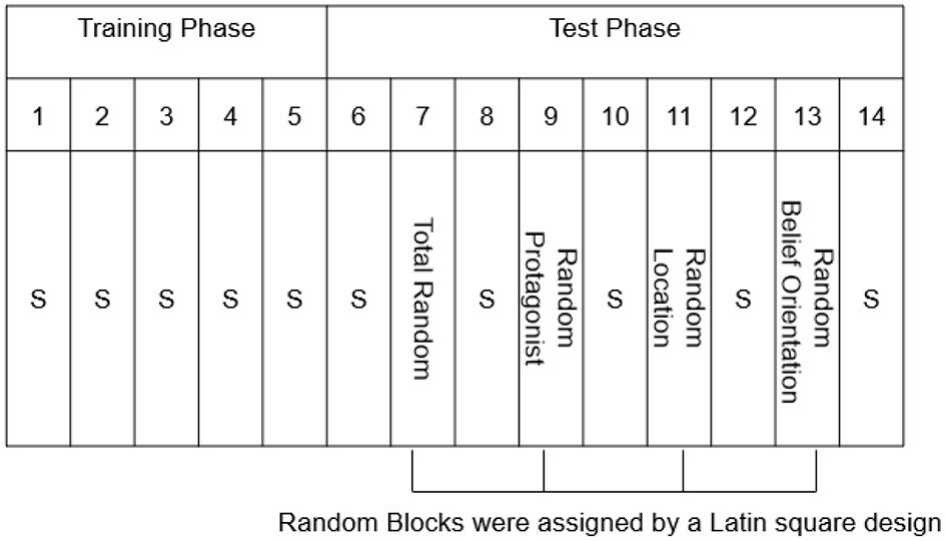
The procedure of Experiment 2 with Blocks numbered 1–14. S = Standard Blocks (with fixed belief-related sequence). Note that every block has a length of 48 trials. One random sequence was created for all dimensions together (i.e., Total Random), and three pseudo-random sequences were created to test implicit learning of each belief-related dimension (i.e., Random Protagonist, Random Belief Orientation, and Random Location). A total of four different orders created by a Latin square design. After the main experiment shown here, there was a recognition test and a post-check questionnaire to measure awareness of the embedded sequence.

Given that implicit learning was robust in Experiment 1, we wanted to explore whether participants could learn more with less training. To do so, we doubled the length of the Standard sequence to 16 trials (Table 1) and shortened the Training Phase. Specifically, the Training Phase consisted of 5 (instead of 6) Standard Blocks. In addition, in the Test Phase, each Random Block was surrounded by one (instead of two) Standard Block (Figure 5, details of Random Blocks see Supplementary Table 2). The four type of random blocks, identical to Experiment 1, were assigned to participants using a Latin square design, to make this order more independent than in Experiment 1. Immediately after the main experiment, we assessed sequence awareness of the location as believed by protagonists in the same way as Experiment 1.

Additionally, during post-check, after the first question asking “Did you notice anything in particular during the experiment” (as in Experiment 1), we added a novel, more specific, post-check question. We told participants that there was a fixed sequence embedded in the task and asked them to report the sequence as much as possible: “Report the order in which papa smurf and smurfette occurred” including whether or not they “could see who gave the flowers” (Social Condition); “Report the order in which the yellow and gray cameras occurred” including whether or not they “focused on you or the flowers” (Camera Condition); “Report the order in which squares and circles appeared” including their colors (Cognitive Condition). All other aspects of the experiment were identical to Experiment 1.

#### Statistical Analysis

First, a general learning effect during the Training Phase was tested by a mixed ANOVA with Training Blocks (1–5) as within-participants factor and the three Conditions (Social, Camera, and Cognitive) as between-participants factor. Second, given our distinct hypothesis with respect to the belief-related dimensions, sequence-specific learning effects during the Test Phase for each dimension (and all dimensions together) was tested separately by a mixed ANOVA with Random vs. Standard Blocks as within-participants factor (using the average of the Standard Blocks adjacent the Random Blocks) and the three Conditions (Social, Camera, and Cognitive) as between-participants factor. Significant main and interaction effects were further tested by a *post-hoc* Bonferroni test.

A sensitivity power analysis (G^*^power 3.1.9.4, Faul et al., 2009) for a mixed ANOVA was applied, using with the same significant level as in Experiment 1 (i.e., alpha significance criterion set to 0.05, standard power criterion set to 80%). The number of groups was set to “3,” and the number of repeated measurements “5” (5 Standard Blocks in the Training Phase) or “2” (Random vs. adjacent Standard Blocks in the Test Phase), indicated that the minimum effect of η^2^ was 0.02 for the general learning effect and 0.03 for the sequence-specific learning effect. Our results showed that the significant findings in Experiment 2 met these effect sizes' requirements. Again, we also used Bayes factors (BF_10_) to interpret our main results.

### Results

The results of three participants in the Social Condition, three participants in the Camera Condition, and five participants in the Cognitive Condition were omitted from the analyses because of excessive error rates (Social = 30%, Camera = 27%, Cognitive = 30%), which were identified as outliers (i.e., above the 3th quartile plus 1.5 times the interquartile range, i.e., 1st vs. 3rd quartile difference). The mean error rates across all blocks for the remaining participants were *M* = 7%, *SD* = 4% (Social); *M* = 9%, *SD* = 5% (Camera); *M* = 7%, *SD* = 4% (Cognitive). Our hypotheses were supported by the following results.

#### General Learning Effect During Training Phase

A mixed ANOVA with Standard Blocks (1–5) as within-participants factor and Condition (Social, Camera, and Cognitive) as between-participants factor was used. For the error rates, there was no general learning effect across Standard Blocks 1–5 (*p* = 0.15). However, there was a main effect of Condition [Social: M = 7%, SD = 5%; Camera: M = 11%, SD = 6%; Cognitive: M = 9%, SD = 5%; *F*_(2,113)_ = 4.58, *MSE* = 8, *p* = 0.01, η^2^ = 0.08, BF_10_ = 10]. *Post-hoc* Bonferroni tests showed that participants made less errors in the Social Condition than in the Camera Condition (*Mean Difference* = *MD* = 4%, *p* < 0.01, BF_10_ = 6,829). The interaction between Condition and Standard Blocks was not significant (*p* > 0.1).

**Figure 6:**
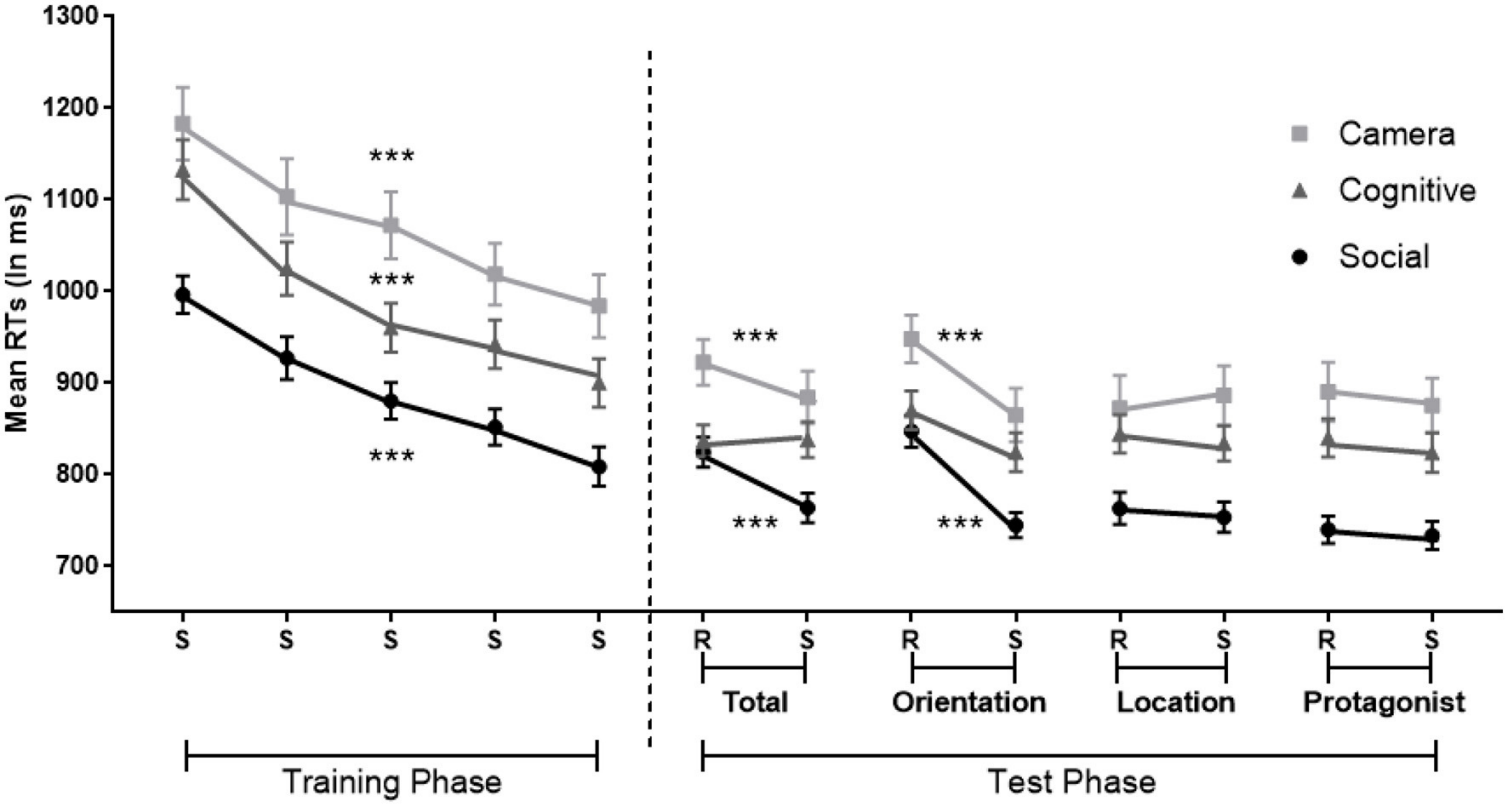
Implicit sequence learning in the Social, Camera and Cognitive Conditions in which the sequence is standard (S) or random (R), with randomization involving orientation (Orientation), the flower's location (Location); the protagonists identity (Protagonist); or these three dimensions together (Total). Orientation involves true-false belief orientation in the Social Condition; current-outdated photos in the Camera Condition and green/blue vs. red/black colors in the Cognitive Condition. Protagonists are Papa Smurf and Smurfette in the Social Condition, gray and yellow cameras in the Camera Condition, and square and circle in the Cognitive Condition. Error bars = Standard errors of the mean. Random blocks of the same dimension and its adjacent sequence blocks were collapsed in the analysis. ***p < 0.001 for significant effects of general and specific implicit learning per condition.

For the RTs (Figure 6 left), the analysis demonstrated a main effect of Standard Blocks 1–5 [*F*_(3.27,368.94)_ = 85.40, *MSE* = 10,540, *p* < 0.001, η^2^ = 0.43, BF_10_ = 4.092e+50], indicating faster responses over training. A main effect of Condition [*F*_(2,113)_ = 11.48, *MSE* = 10,540, *p* < 0.001, η^2^ = 0.17, BF_10_ = 768] was also found. *Post-hoc* tests further showed faster RTs of the Social Condition compared to the Camera and Cognitive Conditions (*MD*_Social−Camera_ = −179 ms, *p* < 0.001, BF_10_ = 1.298e+14, *MD*_Social−Cognitive_ = −99 ms, *p* < 0.05, BF_10_ = 587,938). The interaction between the Condition and Standard Blocks 1–5 was not significant (*p* > 0.5).

#### Sequence-Specific Learning During Test Phase

Four separate mixed ANOVAs with Blocks (one for each type of Random vs. Standard; using the average of the Standard Blocks adjacent the Random Blocks) as within-participants factor and Condition (Social, Camera, and Cognitive) as between-participants factor was applied on error rates and RTs.

For error rates, the mixed ANOVA showed that participants only made more errors in the Random Orientation Blocks vs. Standard Blocks [*F*_(1,113)_ = 9.78, *MSE* = 5, *p* < 0.1, η^2^ = 0.08, BF_10_ = 10], without a significant difference between Conditions (*p* > 0.6). Error rates for other comparisons between Random vs. Standard blocks failed to reach significance (all *ps* > 0.4).

For RTs (Figure 6 right), the analysis on Total Random Blocks revealed a main effect of sequence-specific learning shown by slower responses in the Total Random vs. Standard blocks [*F*_(1,113)_ = 16.81, *MSE* = 3,645, *p* < 0.001, η^2^ = 0.13; BF_10_ = 230]. There was also a main effect of Condition [*F*_(2,113)_ = 7.60, *MSE* = 10,540, *p* = 0.001, η^2^ = 0.12; BF_10_ = 40]. The *post-hoc* Bonferroni tests showed faster RTs in the Social Condition compared to the Camera Condition (*MD*_Social−Camera_ = −109 ms, *p* = 0.001, BF_10_ = 4,494). There was also a significant interaction between sequence-specific learning and Condition [*F*_(2,113)_ = 5.02, *MSE* = 3,645, *p* = 0.008, η^2^ = 0.08, BF_10_ = 4]. A simple effect analysis with Bonferroni correction revealed a sequence-specific learning effect in the Social and Camera Conditions, but not in the Cognitive Condition [Social: *F*_(1,113)_ = 19.77, *p* < 0.001, η^2^ = 0.15; Camera: *F*_(1,113)_ = 8.14, *p* = 0.005, η^2^ = 0.07; Cognitive: *p* = 0.93].

The analysis on Random Orientation Blocks revealed similar results. There was a main effect of sequence-specific learning with slower responses in Random Orientation than the Standard Blocks [*F*_(1,113)_ = 78.19, *MSE* = 4,397, *p* < 0.001 η^2^ = 0.41, BF_10_ = 2.464e+11]. There was also a main effect of Condition [*F*_(2,113)_ = 7.12, *MSE* = 4,397, *p* = 0.001 η^2^ = 0.11, BF_10_ = 32]. The *post-hoc* Bonferroni test on Condition showed faster RTs in the Social Condition compared to the Camera Condition (*MD*_Social−Camera_ = −110 ms, *p* = 0.001, BF_10_ = 2,205). There was also a significant interaction between sequence-specific learning and Condition [*F*_(2,113)_ = 3.52, *MSE* = 4,397, *p* = 0.033, η^2^ = 0.06, BF_10_ = 1]. However, a simple effect analysis with Bonferroni correction further showed a sequence-specific learning effect in all three Conditions [Social: *F*_(1,113)_ = 46.59, *p* < 0.001, η^2^ = 0.29; Camera: *F*_(1,113)_ = 32.17, *p* < 0.001, η^2^ = 0.22; Cognitive: *F*_(1,113)_ = 8.57, *p* = 0.004, η^2^ = 0.07].

The analyses on Random Location and Random Protagonist Blocks showed no significant sequence-specific effects (all *p*s > 0.4), and there was again a main effect of Condition. *Post-hoc* Bonferroni tests showed that participants responded faster in the Social Condition [Random Location: *F*_(2,113)_ = 6.16, *MSE* = 48,960, *p* = <0.01, η^2^ = 0.11, *MD*_Social−Camera_ = −121 ms, *p* < 0.01; Random Protagonist: *F*_(2,113)_ = 10.50, *MSE* = 41,645, *p* < 0.001 η^2^ = 0.16; *MD*_Social−Camera_ = −146 ms, *p* < 0.001, *MD*_Social−Cognitive_ = −95 ms, *p* < 0.02].

**Figure 7:**
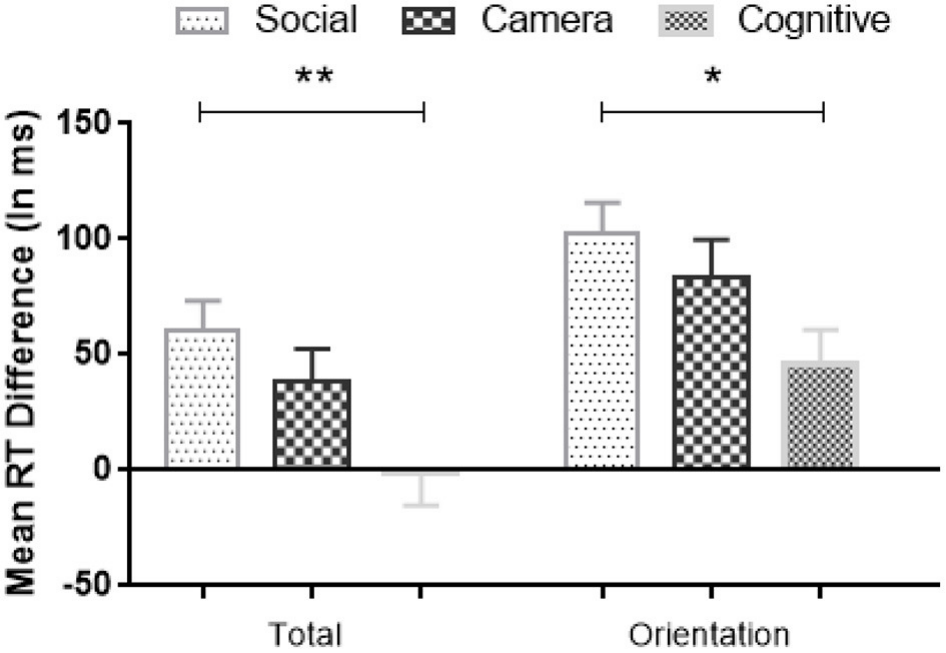
Mean RTs differences (Random minus Standard block) in sequence-specific implicit learning across Conditions; The larger the RT difference score, the stronger the sequence-specific implicit learning effect. Error bars = Standard errors of the mean. * *p* < 0.05; **p < 0.01.

As significant interactions were shown between Condition and the comparison of Random vs. Standard Blocks, we further analyzed the differences in RT between Random and Standard Blocks for all three Conditions. We computed a difference score between Random and Standard Blocks to reflect the amount of sequence learning (Deroost and Soetens, 2006). The larger the RT difference score, the stronger of the sequence-specific learning effect. A one-way ANOVA showed significant difference scores involving the Total Random Blocks [*F*_(2,113)_ = 5.02, *MSE* = 36,588, *p* = 0.008, η^2^ = 0.08, BF_10_ = 5] and the Random Orientation Blocks [*F*_(2, 113)_ = 3.52, *MSE* = 30,953, *p* = 0.033, η^2^ = 0.06, BF_10_ = 1; see Figure 7], but not in the other Random Blocks. *Post-hoc* Bonferroni tests showed that the amount of sequence learning in Social Condition was significantly larger than in the Cognitive Condition (Total Random: *MD*_Social−Cognitive_ = 62 ms, *p* = 0.006, BF_10_ = 22; Random Orientation: *MD*_Social−Cognitive_ = 57 ms, *p* = 0.03, BF_10_ = 7), and numerically stronger than in the Camera Condition, but that latter difference was not significant.

#### Explicit Awareness

After the experiment, conscious awareness of sequences was assessed. In the Social Condition, eight out of the 39 participants reported some sequence awareness (i.e., did “notice anything particular during the experiment”). Of these eight participants, six reported they noticed there was a pattern, two participants noticed something else (e.g., block always ends with papa smurf turned away). As before, we treated all these participants as the “aware” group, whereas the others were treated as the “unaware” group. When participants were informed that there was a sequence, they were asked to “report the order in which Papa Smurf and Smurfette occur[ed] and whether or not they could see who gave the flowers.” The mean length of correct sequence recollection was 4.08/16 for all participants, and 3.50 for the aware group and 4.22 for the unaware group (*t* = −0.94, *p* = 0.35). For the sequence of flowers' location as seen by the smurfs, participants completed the same recognition test as in Experiment 1, in which they were asked to identify a part of all possible flower transitions one-by-one. Responses could be either “yes” or “no.” The average accuracy on the recognition test was 47% which is at chance level (50%). There was no meaningful difference on recognition accuracy for those participants who reported awareness (51%) and those who did not (46%).

Similar results were found in the non-social Conditions. In the Camera Condition, 15 participants reported that they had noticed something. Out of them, nine indicated there was a pattern, and six noticed something else (e.g., always starts with yellow camera). In the Cognitive Condition, 12 participants reported that they had noticed something. Out of them, four reported that they noticed a pattern, six reported that they noticed something else (e.g., sometimes the answers were identical), and two left the answer blank. As before, we treated all these participants as the “aware” group.

When asked to reproduce the sequence, participants' correct recollection was generally very low. For camera colors and orientations, the mean length of correct recollection was 3.32/16 for all participants, with 3.80 for the aware group and 3.04 for the unaware group (*t* = 1.43, *p* = 0.16); for shapes and colors, the mean length of correct recollection was 3.69/16 for all participants, with 4.05 for the aware group and 3.50 for the unaware group (*t* = 0.75, *p* = 0.46). For the location recognition test, mean accuracy was 46% in both non-social Conditions, and there were no significant differences between the aware and unaware groups (43 and 49%, respectively, in the Camera Condition; and 45 and 47%, respectively, in the Cognitive Condition).

We also ran the same mixed ANOVAs for the general and specific learning effects during Training and Testing, now with “Awareness”, (aware vs. unaware) as an additional between-participant factor on RTs. There was no main effect or interaction with Awareness (all *p* > 0.1, see Supplementary Materials). Hence, the learning effect found in Experiment 2 cannot fully due to the awareness.

### Discussion

The main finding of Experiment 2 is that, as hypothesized, participants are able to learn belief sequences in the Social Condition implicitly, independently from their motor responses, although the results are more attenuated than in the Experiment 1. This is unsurprising as mere perceptual implicit learning is more difficult to observe (Kelly and Burton, 2001; Deroost and Soetens, 2006; Coomans et al., 2011). The results of sequence-specific learning in the Test Phase further showed that participants did not learn all dimensions of the sequence, but only the critical true-false belief orientations. Moreover, as hypothesized, the Social Condition led to faster RTs than the non-social Conditions. Perhaps more importantly, there was a stronger sequence specific learning effect in the Social Condition, especially in the critical Orientation dimension. This suggests that implicit social sequence learning which requires the inferring of the mental states of other protagonists, might differ in some critical aspects from implicit non-social sequence learning processes. We return to this issue in the general discussion.

## General Discussion

The current research investigated the possibility of implicit learning of belief-related sequences in a social context. We developed a novel belief SRT task by combining elements from a classic false belief and a serial reaction time task. In the present task, unbeknownst to the participants, various belief dimensions were embedded, such as the different identity of protagonists (who holds the belief), their true or false belief orientation (whether their beliefs are true or false, i.e., looking toward or away from the flowers), and flower location believed by protagonists. As hypothesized, the results suggest that sequences were implicitly learned, that is, with little explicit awareness, especially about true-false belief orientations.

To recapitulate briefly, in Experiment 1, the results clearly showed that sequence knowledge was implicitly acquired on all three belief-related dimensions. This is generally consistent with previous studies on implicit learning of multiple sequences (Mayr, 1996; Deroost and Soetens, 2006). This supports our hypothesis that people can learn sequential knowledge on false and true beliefs in a social context. However, one important shortcoming of Experiment 1 was that the motor response was confounded with the embedded belief-related sequence on flower location. Consequently, participants may have learned the sequence of flower locations because they were aided by the implicit learning of the parallel motor sequences (Deroost and Soetens, 2006), thus leaving more resources to learn the other dimensions of the standard sequence as well. To investigate implicit belief learning while minimizing a motor confound, in Experiment 2, participants had to indicate how many flowers the protagonists believed to have received, which required a response that was independent from the embedded sequence. This enabled us to rule out potential motor learning effects. As previous research indicated, perceptual sequence learning is vulnerable to stimulus complexity and difficult to demonstrate compared to motor learning (Kelly and Burton, 2001; Coomans et al., 2011). Notwithstanding the frugality of this process, Experiment 2 demonstrated implicit sequence learning of true-false belief orientations in a social context.

This study is the first evidence demonstrating that people can implicitly learn repeated belief-related sequences, in particular, sequences of false and true belief orientations. This is a crucial ability whereby people come to learn intuitively the stable regularities in dynamic social stimuli, which may help them to anticipate behaviors of others and the consequences for themselves, and to recognize deviations which can modify future interactions. This is a novel finding that may provide an important proof-of-principle on people's ability to learn and use repeated patterns of behavioral cues implicitly, such as true-false belief orientation, to predict the next sequences of others' beliefs. This skill likely facilitates interaction and cooperation between individuals, much like how dance partners or office colleagues perform more efficiently by implicitly predicting their own and their partner's moves or beliefs. Note that the present study focused on implicit learning of belief sequences, not on implicit inferences of beliefs. On the contrary, belief inferences were explicitly requested by instruction, and the instruction detailed how true and false beliefs had to be deduced. Hence, this study has no implications for the ongoing debate about the reliability of implicit mentalizing (Schneider et al., 2017; Poulin-Dubois et al., 2018).

It is interesting to observe that implicit learning of the distinct protagonists holding these beliefs, took place systemically only when implicit learning was aided by motor responses in Experiment 1, and not when learning was purely perceptual in Experiment 2. Learning to anticipate which protagonist would hold specific beliefs, might have facilitated responses nonetheless. We surmise that this learning effect is less robust because detecting and anticipating true vs. false beliefs is more essential for providing correct responses to the task. It is also possible that doubling the length of the standard sequence (16 instead of eight trials) and reducing its learning across the training and test phases in Experiment 2, may have contributed to this. Consequently, less processing resources were available for other relevant information on the protagonists and their beliefs.

An important novelty of Experiment 2 was that two non-social Conditions were included by replacing the two protagonists with two non-social objects: colored cameras or geometric shapes. This enabled us to contrast and identify social belief processes in comparison with purely cognitive processes. Although participants learned the orientation sequences in the three Conditions, the results revealed that participants generally responded faster in the Social Condition than the non-social Camera and Cognitive Conditions across all Phases. More importantly, the sequence-specific learning effect was most pronounced in the Test Phase during implicit learning of the true-false orientation sequence (i.e., greatest slowing down of responses when the Standard orientation sequence was interrupted by a Random sequence), although this effect was only significant compared to the Cognitive Condition. The lack of significant differences with the Camera Condition might be due to some participants who may have anthropomorphized the cameras and their orientation, which is less likely to happen in the Cognitive Condition. It might also be due to a similar load in the Social and Camera Conditions for remembering and accessing smurfs and cameras' orientations, as opposed to a higher load for color-defined orientations with artificial rules in the Cognitive Condition. On the other hand, the fact that the same pattern of results was obtained in the Social and Camera Conditions might indicate that the advantage in the Social Condition is perhaps not due to the attribution of false vs. true beliefs, but rather to how social schemata are so overlearned that participants can easily integrate the instruction and related memorization (i.e., remembering the prior true trial of a protagonist) in an adequate response. Still, it is very interesting that RTs are generally faster—at least numerically—in the Social vs. non-social Camera and Cognitive Conditions.

The faster responses and stronger implicit learning effects overall in the Social Condition are consistent with previous studies without a SRT framework, showing faster reaction times to social mental representations (e.g., beliefs) than to non-social representations (e.g., photos and arrows; Cohen and German, 2010; Callejas et al., 2011). Cohen and German (2010) reported that participants responded faster to probes suggesting the location of a hidden object, when the probe involved someone's belief rather than an arrow on a map. Likewise, Callejas et al. (2011) found faster responses on comprehension questions involving false belief than outdated photos. Perhaps more telling is the study by Saxe et al. (2006), mentioned earlier, which investigated an analogous false belief design with animations. In their social and non-social Conditions, the participants read structurally similar instructions—Social: “where does the girl think the chocolate is? If she is looking away, she only saw it go into the first box. However, if she is facing the boxes, then she saw it go into the last box”; Non-social: “If the girl is facing the boxes at the end of the trial, press the button for the last box. If the girl is looking away from the boxes, press the button for the first box.” Despite the structural equivalence of the task, the authors found faster RTs for inferring the girl's belief than for following the non-social rule instruction.

One explanation for faster RTs in the Social Condition, mentioned earlier, is the extensive naturalistic practice with social mentalizing in daily life. This echoes similar findings in reasoning where pragmatic reasoning schemas from everyday life in comparison to abstract rules, can facilitate deductive reasoning (Wason and Shapiro, 1971; Cheng and Holyoak, 1985). Conversely, artificial and purely cognitive learning typically starts at school and is further applied at work, and is often applied and practiced in these environments only. As suggested by bib4 (2011, p. 1), “extensive naturalistic practice with ToM reasoning may enable a more flexible and efficient mental representation of false belief stories.” The naturalistic mentalizing practice may have speeded up the responses, leaving more cognitive resources to discover the sequential structure in the social condition as opposed to the non-social conditions (Deroost and Soetens, 2006).

Another explanation for the superior performance in the Social Condition may stem from “domain specific mechanisms within human cognition for encoding and reasoning about mental states” (Cohen and German, 2010, p. 417). Neuroimaging studies provide some initial evidence that social processing activates cortical areas preferentially recruited for mentalizing (e.g., the temporal-parietal junction), in particular for taking the perspective of others' beliefs (Saxe et al., 2006; Döhnel et al., 2012).

It remains possible that the slower processing in the non-social Conditions is due to the difficulty of remembering the response rules, especially for the Cognitive Condition (i.e., remembering and retrieving artificial rules of colored shapes). However, the slowest responses were revealed in the Camera Condition, where we used traffic cameras and photos, which are familiar to most people. Although we do not have an explanation for the slowest responses in that Condition, it is unlikely that people find it most difficult to identify and remember the rules in the camera Condition, as people use cameras in daily life and are, therefore, familiar with the reality that when the camera is turned away from a flower, a photo cannot be taken of that flower.

### Limitations

Although we found evidence for implicit sequence learning in a social context, the present results do not allow us to identify precisely what strategy participants used to learn the sequences or which part of the sequences they actually learned. Perhaps implicit learning in the current study was driven by some kind of more simple transitions and expectation? For example, the expectation that after each true or false trial, the other type of trial would follow within one of the next two steps of the belief sequence? That no sequence of three consecutive trials of the same belief type ever occurred (which was indeed the case)? Note that similar basic restrictions were also built in Total Random blocks, and thus provide no explanation for its somewhat weaker effect than Random Orientation. Perhaps sequence learning was driven by another kind of implicit rule that we are currently unaware of. This is an issue for future research.

Another limitation of the present study refers to the explicit knowledge assessment procedure. We presented a recognition task for location, but not for the protagonists' orientation. This was only done on paper in Experiment 2, by requesting to reproduce the standard sequence as much as possible. Moreover, to be sensitive to any hint of explicit knowledge, a recognition task should include as much elements of the original task to recreate and prime the original learning context as much as possible (Destrebecqz and Cleeremans, 2001), such as repeating the task itself for a limited number of trials, and asking whether the sequence was familiar or not. This is certainly a valuable contribution for future research.

The present experiments used popular symbolic figures and objects as stimuli, such as smurfs, cameras and colorful shapes, to increase the generality of our material and to reduce the difficulty or any other potential influence on implicit learning from cultural or socio-economic differences in our samples. Since we did not measure individual capacities for mentalizing, we do not know either to what extent the ability to mentalize might also impact on implicit learning of belief sequences. The effects of mentalizing ability on social sequence learning is an important issue for future research. Moreover, in the present experiments, participants were explicitly told to infer protagonists' beliefs. Although implicit mentalizing is beyond the scope of the present study, it might inspire future studies to investigate the processes of implicit tracking sequential beliefs without explicit motivations. This might provide answer to questions such as: If participants do not get instructions, would they automatically understand the meaning of orientations?

### Implications

Although there are many classic SRT studies investigating sequence learning, these were mostly focused on invariant predictable patterns in an artificial cognitive context. Although we added social elements into our SRT task, the belief orientation sequence was still very restricted compared to real social life which is always changing along multiple factors. For example, a fluent social interaction often relies on facial, gestural and vocal sequences which constitute a much more complex array of stimuli than a single-dimensional sequence in an SRT task (Zwart et al., 2018). Nonetheless, the present investigation of implicit sequence learning for several belief dimensions is a first important step to increase our current scientific understanding of the implicit nature of advanced social learning and reasoning.

Neuroimaging evidence suggests that the temporo-parietal junction (TPJ) is a crucial cortical area that is involved in mentalizing processes, especially in belief reasoning (Van Overwalle and Baetens, 2009; Cohen and German, 2010; Kampis et al., 2017). More importantly, recent studies showed that the cerebellum also contributes to social processes by detecting repetitive patterns (Van Overwalle et al., 2020). In line with of idea of social and non-social domain-specific mechanisms (Cohen and German, 2010), future research using neuroimaging might provide more convincing evidence on the specific processes underlying social vs. non-social sequence learning.

At the clinical level, implicit sequence learning in a social context can contribute to a better understanding of social impairments in patients, so that we can better tailor social training programs to the specific needs of these populations. For example, although implicit sequence learning of people with autism seems to be intact (Brown et al., 2010), they require increased training for detecting invariant visual structures (Gordon and Stark, 2007), and sometimes fail to detect invariant patterns entirely, for example in social communication (Hellendoorn et al., 2015). This is likely because communication may involve multiple sequences such as speakers' voices, complex syntax (Lee et al., 2018), and complex structure in narratives (McCabe et al., 2013). It is still an open question to what extent individuals with autism are impaired in implicit learning of regularities in social beliefs. The present implicit social sequence learning task may provide novel insights and tools to identify such social deficiencies. In particular, it might be a more efficient way to diagnose social capacities of individuals with social impairments, as this task is tailored to their specific limitations. This is an important avenue for future research.

### Conclusion

The present study demonstrates the capacity of implicit learning of belief-related sequences, and in particular, the learning of sequences of true and false belief orientations. Moreover, compared to non-social conditions, we found a clear advantage in processing speed and implicit learning for social belief state reasoning. At the clinical level, the present task can give directions for identifying impairments in social sequence learning, such as in individuals with autism.

## Data Availability Statement

The raw data supporting the conclusions of this article will be made available by the authors, without undue reservation.

## Ethics Statement

The studies involving human participants were reviewed and approved by Vrije Universiteit Brussel. The patients/participants provided their written informed consent to participate in this study.

## Author Contributions

QM, FV, EH, GF, and MP contribute to the experiment design. QM and MP contribute to data analysis. QM, FV, KB, and ND contribute to the manuscript. All authors contributed to the article and approved the submitted version.

## Conflict of Interest

The authors declare that the research was conducted in the absence of any commercial or financial relationships that could be construed as a potential conflict of interest.
