# Supplementary material for: Implicit Learning of True and False Belief Sequences
Source: Front Psychol. 2021 Mar 26;12:643594. doi: 10.3389/fpsyg.2021.643594 (PMC8032999; doi:10.3389/fpsyg.2021.643594)
Supplement: Supplementary file 1 [file Table_1.DOCX]

**Supplementary materials**

***Effect of Awareness in Experiment 1***

To investigate whether the sequence learning effect was modulated by explicit awareness, we reran the previous RT analyses. The results revealed that participants benefitted little from their explicit knowledge.

For the general learning effect (i.e., slowing down of RTs over Blocks), we ran a mixed-ANOVA with Standard Blocks (1 – 6), and additionally Awareness (aware versus unaware) as between-participant factor. The main effect of Standard Blocks (1 - 6) remained the same (*p* < 0.001, *η^2^* =0.59), and there was no main effect of Awareness (*p* = 0.07), or their interaction (*p* = 0.22).

For each sequence-specific learning effect, we ran a mixed ANOVA with Block (Random versus Standard), and additionally Awareness (aware versus unaware) as between-participant factor. There was no change in the significant effects of the comparison between Random versus Standard Blocks (all *p* < 0.05, *η^2^* > 0.25), and there was no main effect of Awareness (all *p* > 0.1) or their interaction (all *p* > 0.5).

***Effect of Awareness in Experiment 2***

As in Experiment 1, we reran the previous RT analyses. The results showed that awareness did not significantly modulate sequence learning effects.

For the general learning effect (i.e., slowing down of RTs over Blocks), we ran a mixed-ANOVA with Standard Blocks 1 – 5 as within-participant factor, and Condition and additionally Awareness (aware versus unaware) as between-participant factors. The main effects of the Standard Blocks 1 – 5 (*F* _(3.31, 364.63)_ = 78.15, *MSE =* 33398*,* *p* < 0.001, *η^2^* =0.42) and the Conditions (*F* _(1, 110)_ = 9.35, *MSE =* 137230*,* *p* < 0.001, *η^2^* =0.15) remained the same. There were no interactions between the Standard Blocks 1 – 5 and the Conditions (*p* > 0.1) and between Awareness and the Conditions (*p* > 0.05). However, there was an interaction between the Standard Blocks 1 – 5 and Awareness (*F* _(3.31, 364.63)_ = 2.67, *MSE =* 33398*,* *p* < 0.05, *η^2^* =0.02). This result showed that participants who had sequential awareness had slower RTs at the beginning but faster RTs at the end of the Training Phase.

For each sequence-specific learning effect, we ran a mixed ANOVA with Block (Random versus Standard) as within-participant factor, and Condition and additionally Awareness (aware versus unaware) as between-participant factor. Again, we found sequence-specific learning effect in the comparisons of Total Random/Random Orientation Blocks and their adjacent Standard Blocks.

Results revealed a main effect indicating slower responses in the Total Random Block versus Standard Blocks (*F* _(1, 110)_ = 12.52, *MSE* = 3470, *p* < 0.01, *η^2^* = 0.10), a main effect of the Conditions showing faster responses in the Social Condition (*F* _(2, 110)_ = 5.87, *MSE* = 31864, *p* < 0.01, *η^2^* = 0.10). There was no main effect of Awareness (*p* > 0.6), and there were no significant interactions between specific learning effect in Total Random and Awareness (*p* > 0.4) and between Conditions and Awareness (*p* > 0.2).

The same results were found for the Random Orientation Block, with slower responses than in the Standard Blocks (*F* _(1, 110)_ = 59.81, *MSE* = 4189, *p* < 0.001 *η^2^* = 0.35), a main effect of Condition showing faster responses in the Social Condition (*F* _(2, 110)_ = 5.85, *MSE* = 33412, *p* < 0.01, *η^2^* = 0.10). There was no main effect of Awareness (*p* > 0.2), and there were no significant interactions between sequence-specific learning effect in the Random Orientation block and Awareness (*p* > 0.7) and between the Conditions and Awareness (*p* > 0.1).

**Table S1:** Standard sequence and pseudo-random sequences of the three dimensions; Experiment 1.

| Standard Sequence to be learned (repeated every 8 trials) | | | | | | | | | | | | | | | | |
| --- | --- | --- | --- | --- | --- | --- | --- | --- | --- | --- | --- | --- | --- | --- | --- | --- |
| Location | 1 | 1 | 4 | 3 | 4 | 3 | 2 | 2 |  |  |  |  |  |  |  |  |
| Protagonist | M | M | Fe | M | Fe | M | Fe | Fe |  |  |  |  |  |  |  |  |
| Belief Orientation | T | Fa | T | T | Fa | Fa | T | Fa |  |  |  |  |  |  |  |  |
| Prior true trial |  | -1 |  |  | -2 | -2 |  | -1 |  |  |  |  |  |  |  |  |
| Random Locations (Motor Response; repeated every 16 trials) | | | | | | | | | | | | | | | | |
| Location | 4 | 4 | 1 | 2 | 1 | 2 | 3 | 3 | 2 | 2 | 3 | 1 | 3 | 1 | 4 | 4 |
| Protagonist | M | M | Fe | M | Fe | M | Fe | Fe | M | M | Fe | M | Fe | M | Fe | Fe |
| Belief Orientation | T | Fa | T | T | Fa | Fa | T | Fa | T | Fa | T | T | Fa | Fa | T | Fa |
| Prior true trial |  | -1 |  |  | -2 | -2 |  | -1 |  | -1 |  |  | -2 | -2 |  | -1 |
| Random Belief Orientations (repeated every 8 trials) | | | | | | | | | | | | | | | | |
| Location | 1 | 1 | 4 | 3 | 4 | 3 | 2 | 2 |  |  |  |  |  |  |  |  |
| Protagonist | M | Fe | Fe | M | Fe | M | Fe | M |  |  |  |  |  |  |  |  |
| Belief Orientation | T | T | T | T | Fa | Fa | T | T |  |  |  |  |  |  |  |  |
| Prior true trial |  |  |  |  | -2 | -2 |  |  |  |  |  |  |  |  |  |  |
| Random Protagonists (repeated every 16 trials) | | | | | | | | | | | | | | | | |
| Location | 1 | 1 | 4 | 3 | 4 | 3 | 2 | 2 | 1 | 1 | 4 | 3 | 4 | 3 | 2 | 2 |
| Protagonist | Fe | Fe | Fe | M | Fe | M | M | M | Fe | Fe | M | Fe | M | Fe | M | M |
| Belief Orientation | T | Fa | T | T | Fa | Fa | T | Fa | T | Fa | T | T | Fa | Fa | T | Fa |
| Prior true trial |  | -1 |  |  | -2 | -2 |  | 1 |  | -1 |  |  | -2 | -2 |  | 1 |

Note: M = male, Fe = female, T = true, Fa = false. The Random sequences in the table were created by pseudo-random orders of one dimension of the Standard sequence (i.e., Location, Belief Orientation or Protagonist), while keeping intact the two other dimensions. Note, however, that the Random Belief Orientation inevitably also induced changes in the sequence of the Protagonist. These pseudo-random orders were further constrained to contain about the same number of trials between a false and (previous) true trials (as indicated by “Prior true trial”) and the number of subsequent trials with the same true or false Belief Orientation which should not exceed 2 (as in the Standard sequence). Some Random sequences were made twice as long to increase unpredictability. For Total Random blocks (not shown), all dimensions were totally randomized with the limitation of at most 2 subsequent trials of the same true or false Belief Orientation, consistent with the Standard sequence.

**Table S2:** Standard sequence and pseudo-random sequences of the three dimensions; Experiment 2.

| Standard Sequence to be learned (repeated every 16 trials) | | | | | | | | | | | | | | | | |
| --- | --- | --- | --- | --- | --- | --- | --- | --- | --- | --- | --- | --- | --- | --- | --- | --- |
| Location | 1 | 1 | 4 | 3 | 4 | 3 | 2 | 2 | 2 | 1 | 1 | 4 | 3 | 4 | 3 | 2 |
| Protagonist | M | M | Fe | M | Fe | M | Fe | Fe | Fe | Fe | Fe | M | Fe | M | Fe | M |
| Belief Orientation | T | Fa | T | T | Fa | Fa | T | Fa | Fa | T | Fa | T | T | Fa | Fa | T |
| Prior true trial |  | -1 |  |  | -2 | -2 |  | -1 | -2 |  | -1 |  |  | -2 | -2 |  |
| Random Locations (repeated every 48 trials) | | | | | | | | | | | | | | | | |
| Location | 4 | 4 | 3 | 1 | 3 | 1 | 3 | 3 | 3 | 2 | 2 | 1 | 2 | 1 | 2 | 4 |
| Protagonist | M | M | Fe | M | Fe | M | Fe | Fe | Fe | Fe | Fe | M | Fe | M | Fe | M |
| Belief Orientation | T | Fa | T | T | Fa | Fa | T | Fa | Fa | T | Fa | T | T | Fa | Fa | T |
| Prior true trial |  | -1 |  |  | -2 | -2 |  | -1 | -2 |  | -1 |  |  | -2 | -2 |  |
| (continued: trials 17 - 32) | | | | | | | | | | | | | | | | |
| Location | 3 | 3 | 2 | 1 | 2 | 1 | 4 | 4 | 4 | 3 | 3 | 4 | 2 | 4 | 2 | 1 |
| Protagonist | M | M | Fe | M | Fe | M | Fe | Fe | Fe | Fe | Fe | M | Fe | M | Fe | M |
| Belief Orientation | T | Fa | T | T | Fa | Fa | T | Fa | Fa | T | Fa | T | T | Fa | Fa | T |
| Prior true trial |  | -1 |  |  | -2 | -2 |  | -1 | -2 |  | -1 |  |  | -2 | -2 |  |
| (continued: trials 33 - 48) | | | | | | | | | | | | | | | | |
| Location | 2 | 2 | 3 | 4 | 3 | 4 | 1 | 1 | 1 | 4 | 4 | 3 | 1 | 3 | 1 | 2 |
| Protagonist | M | M | Fe | M | Fe | M | Fe | Fe | Fe | Fe | Fe | M | Fe | M | Fe | M |
| Belief Orientation | T | Fa | T | T | Fa | Fa | T | Fa | Fa | T | Fa | T | T | Fa | Fa | T |
| Prior true trial |  | -1 |  |  | -2 | -2 |  | -1 | -2 |  | -1 |  |  | -2 | -2 |  |
| Random Belief Orientations (repeated every 48 trials) | | | | | | | | | | | | | | | | |
| Location | 1 | 1 | 4 | 3 | 4 | 3 | 2 | 2 | 2 | 1 | 1 | 4 | 3 | 4 | 3 | 2 |
| Protagonist | M | M | Fe | M | Fe | M | Fe | Fe | Fe | Fe | Fe | M | Fe | M | Fe | M |
| Belief Orientation | T | T | T | T | Fa | Fa | T | Fa | T | T | T | T | T | Fa | Fa | T |
| Prior true trial |  |  |  |  | -2 | -2 |  | -1 |  |  |  |  |  | -2 | -2 |  |
| (continued: trials 17 - 32) | | | | | | | | | | | | | | | | |
| Location | 1 | 1 | 4 | 3 | 4 | 3 | 2 | 2 | 2 | 1 | 1 | 4 | 3 | 4 | 3 | 2 |
| Protagonist | M | M | Fe | M | Fe | M | Fe | Fe | Fe | Fe | Fe | M | Fe | M | Fe | M |
| Belief Orientation | T | T | T | T | Fa | Fa | T | T | Fa | T | Fa | T | T | Fa | Fa | T |
| Prior true trial |  |  |  |  | -2 | -2 |  |  | -1 |  | -1 |  |  | -2 | -2 |  |
| (continued: trials 33 - 48) | | | | | | | | | | | | | | | | |
| Location | 1 | 1 | 4 | 3 | 4 | 3 | 2 | 2 | 2 | 1 | 1 | 4 | 3 | 4 | 3 | 2 |
| Protagonist | M | M | Fe | M | Fe | M | Fe | Fe | Fe | Fe | Fe | M | Fe | M | Fe | M |
| Belief Orientation | T | Fa | T | T | Fa | Fa | T | T | T | T | Fa | T | T | Fa | Fa | T |
| Prior true trial |  | -1 |  |  | -2 | -2 |  |  |  |  | -1 |  |  | -2 | -2 |  |
| Random Protagonists (repeated every 48 trials) | | | | | | | | | | | | | | | | |
| Location | 1 | 1 | 4 | 3 | 4 | 3 | 2 | 2 | 2 | 1 | 1 | 4 | 3 | 4 | 3 | 2 |
| Protagonist | Fe | Fe | M | Fe | M | Fe | M | M | M | Fe | Fe | Fe | M | Fe | M | Fe |
| Belief Orientation | T | Fa | T | T | Fa | Fa | T | Fa | Fa | T | Fa | T | T | Fa | Fa | T |
| Prior true trial |  | -1 |  |  | -2 | -2 |  | -1 | -2 |  | -1 |  |  | -2 | -2 |  |
| (continued: trials 17 - 32) | | | | | | | | | | | | | | | | |
| Location | 1 | 1 | 4 | 3 | 4 | 3 | 2 | 2 | 2 | 1 | 1 | 4 | 3 | 4 | 3 | 2 |
| Protagonist | Fe | Fe | Fe | M | Fe | M | M | M | M | M | M | Fe | M | Fe | M | Fe |
| Belief Orientation | T | Fa | T | T | Fa | Fa | T | Fa | Fa | T | Fa | T | T | Fa | Fa | T |
| Prior true trial |  | -1 |  |  | -2 | -2 |  | -1 | -2 |  | -1 |  |  | -2 | -2 |  |
| (continued: trials 33 - 48) | | | | | | | | | | | | | | | | |
| Location | 1 | 1 | 4 | 3 | 4 | 3 | 2 | 2 | 2 | 1 | 1 | 4 | 3 | 4 | 3 | 2 |
| Protagonist | M | M | M | Fe | M | Fe | Fe | Fe | Fe | M | M | M | Fe | M | Fe | M |
| Belief Orientation | T | Fa | T | T | Fa | Fa | T | Fa | Fa | T | Fa | T | T | Fa | Fa | T |
| Prior true trial |  | -1 |  |  | -2 | -2 |  | -1 | -2 |  | -1 |  |  | -2 | -2 |  |

Note: M = male, Fe = female, T = true, Fa = false. The Random sequences in the table were created by pseudo-random orders of Location, Belief Orientation and Protagonist as explained in Supplementary Table S1. Random sequences were made three times as long to increase unpredictability. For total random blocks (not shown), all dimensions were totally randomized with the limitation of at most 2 subsequent trials of the same true or false type, consistent with the Standard sequence.

**Table S3**: RTs reflecting General learning (training phase) and Sequence-specific learning (test phase); Experiment 1

|  | Training Phase | | Test Phase | | | | | | | |
| --- | --- | --- | --- | --- | --- | --- | --- | --- | --- | --- |
|  |  | | Total | | Belief Orientation | | Location | | Protagonists | |
|  | Block 1 | Block 6 | Random | Standard | Random | Standard | Random | Standard | Random | Standard |
| M | 1060 | 599 | 1028 | 545 | 461 | 271 | 820 | 379 | 450 | 389 |
| SD | 183 | 313 | 211 | 222 | 295 | 184 | 161 | 217 | 226 | 212 |

Note: Block 1 is the first block in the Training Phase, Block 6 is the last block in the Training Phase. The Test Phase involved Random Blocks and adjacent Standard Blocks, which were collapsed in the analysis.

**Table S4**: RTs reflecting General learning (training phase) and Sequence-specific learning (test phase); Experiment 2

|  |  | Training Phase | | Test Phase | | | | | | | |
| --- | --- | --- | --- | --- | --- | --- | --- | --- | --- | --- | --- |
|  |  |  | | Total | | Orientation | | Location | | Protagonists | |
|  |  | Block 1 | Block 5 | Random | Standard | Random | Standard | Random | Standard | Random | Standard |
| Social | *M* | 997 | 809 | 825 | 764 | 848 | 745 | 763 | 754 | 740 | 734 |
|  | *SD* | 128 | 133 | 102 | 100 | 110 | 84 | 110 | 104 | 93 | 96 |
| Camera | *M* | 1183 | 984 | 923 | 885 | 948 | 865 | 873 | 887 | 891 | 876 |
|  | *SD* | 254 | 221 | 160 | 184 | 166 | 187 | 229 | 208 | 207 | 191 |
| Cognitive | *M* | 1133 | 196 | 837 | 838 | 870 | 825 | 845 | 834 | 840 | 824 |
|  | *SD* | 900 | 159 | 105 | 117 | 127 | 128 | 127 | 116 | 126 | 131 |

Note: Block 1 is the first block in the Training Phase, Block 5 is the last block in the Training Phase. The Test Phase involved Random Blocks and adjacent Standard Blocks, which were collapsed in the analysis. Orientation involves true-false belief orientation in the Social condition; current-outdated photos in the Camera condition and green/blue versus red/black colors in the Control condition. Protagonists are Papa Smurf and Smurfette in the Social condition, grey and yellow cameras in the Camera condition, and square and circle shapes in the Control condition.
