# Supplementary material for: Implicit Learning of True and False Belief Sequences
Source: Front Psychol. 2021 Mar 26;12:643594. doi: 10.3389/fpsyg.2021.643594 (PMC8032999; doi:10.3389/fpsyg.2021.643594)
Supplement: Supplementary file 2 [file Presentation_1.PPTX]

## Slide 1
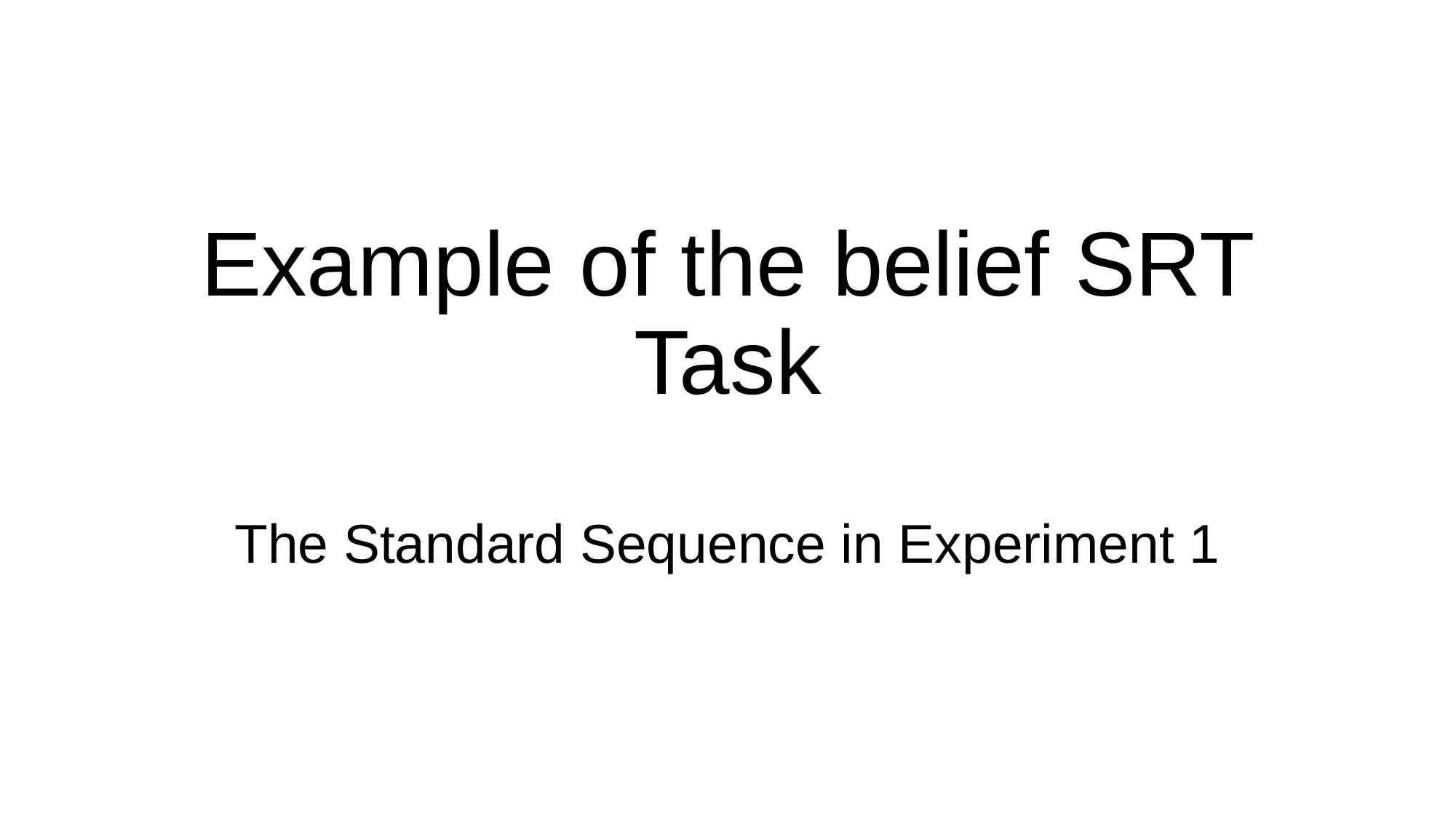

# Example of the belief SRT Task
The Standard Sequence in Experiment 1

## Slide 2
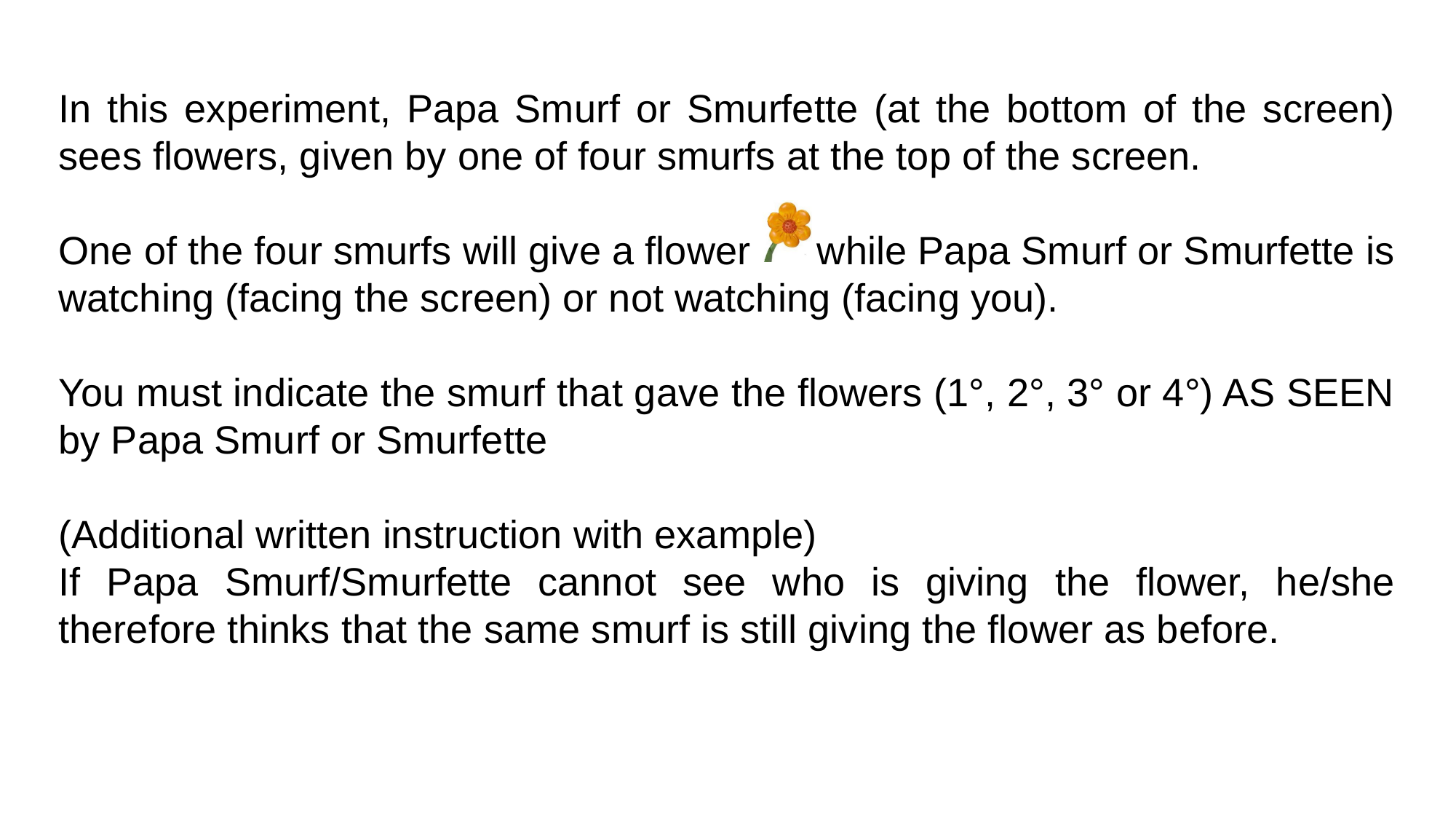

In this experiment, Papa Smurf or Smurfette (at the bottom of the screen) sees flowers, given by one of four smurfs at the top of the screen.
One of the four smurfs will give a flower while Papa Smurf or Smurfette is watching (facing the screen) or not watching (facing you).
You must indicate the smurf that gave the flowers (1°, 2°, 3° or 4°) AS SEEN by Papa Smurf or Smurfette
(Additional written instruction with example)
If Papa Smurf/Smurfette cannot see who is giving the flower, he/she therefore thinks that the same smurf is still giving the flower as before.

## Slide 3
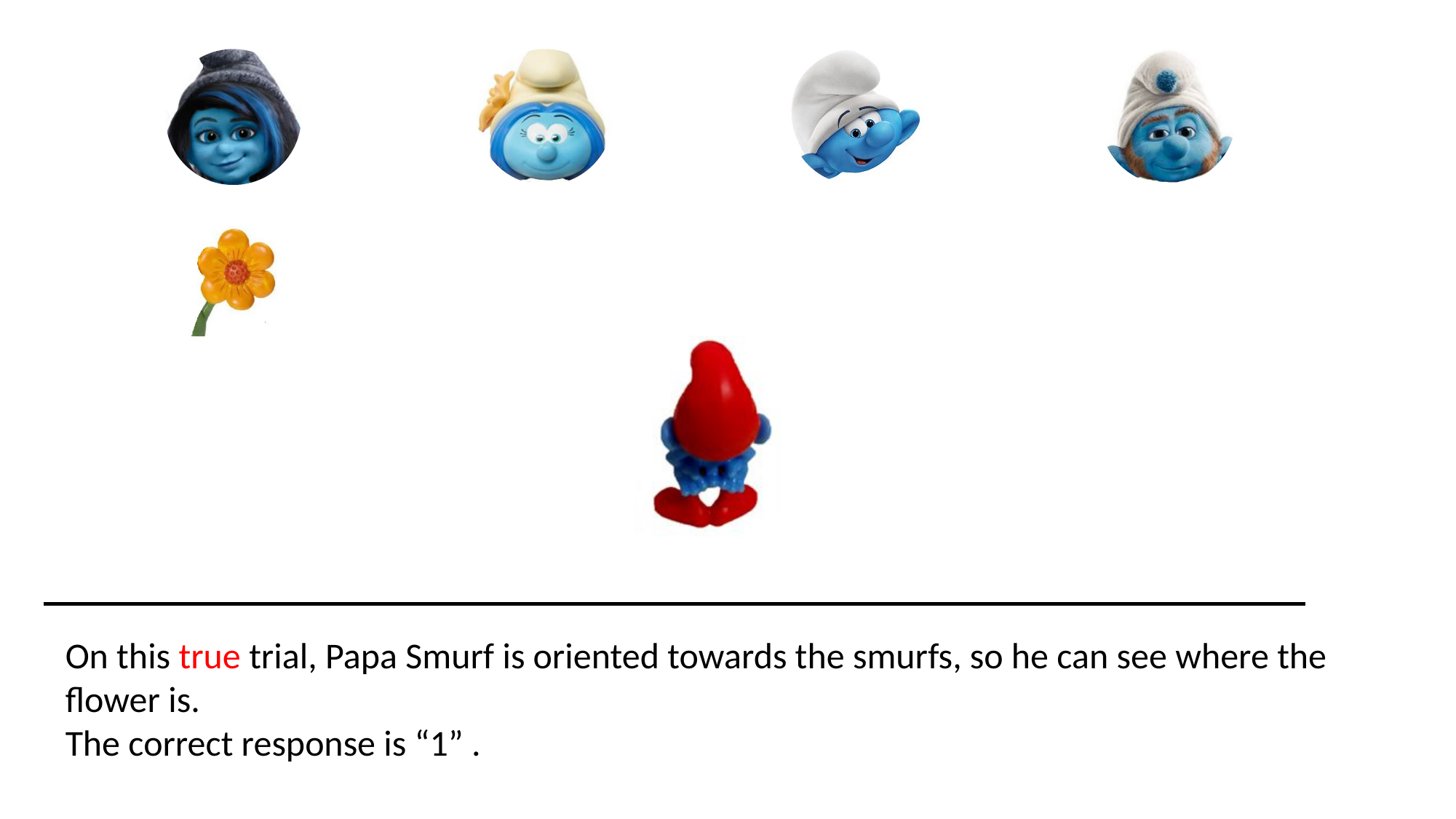

On this true trial, Papa Smurf is oriented towards the smurfs, so he can see where the flower is.
The correct response is “1” .

## Slide 4
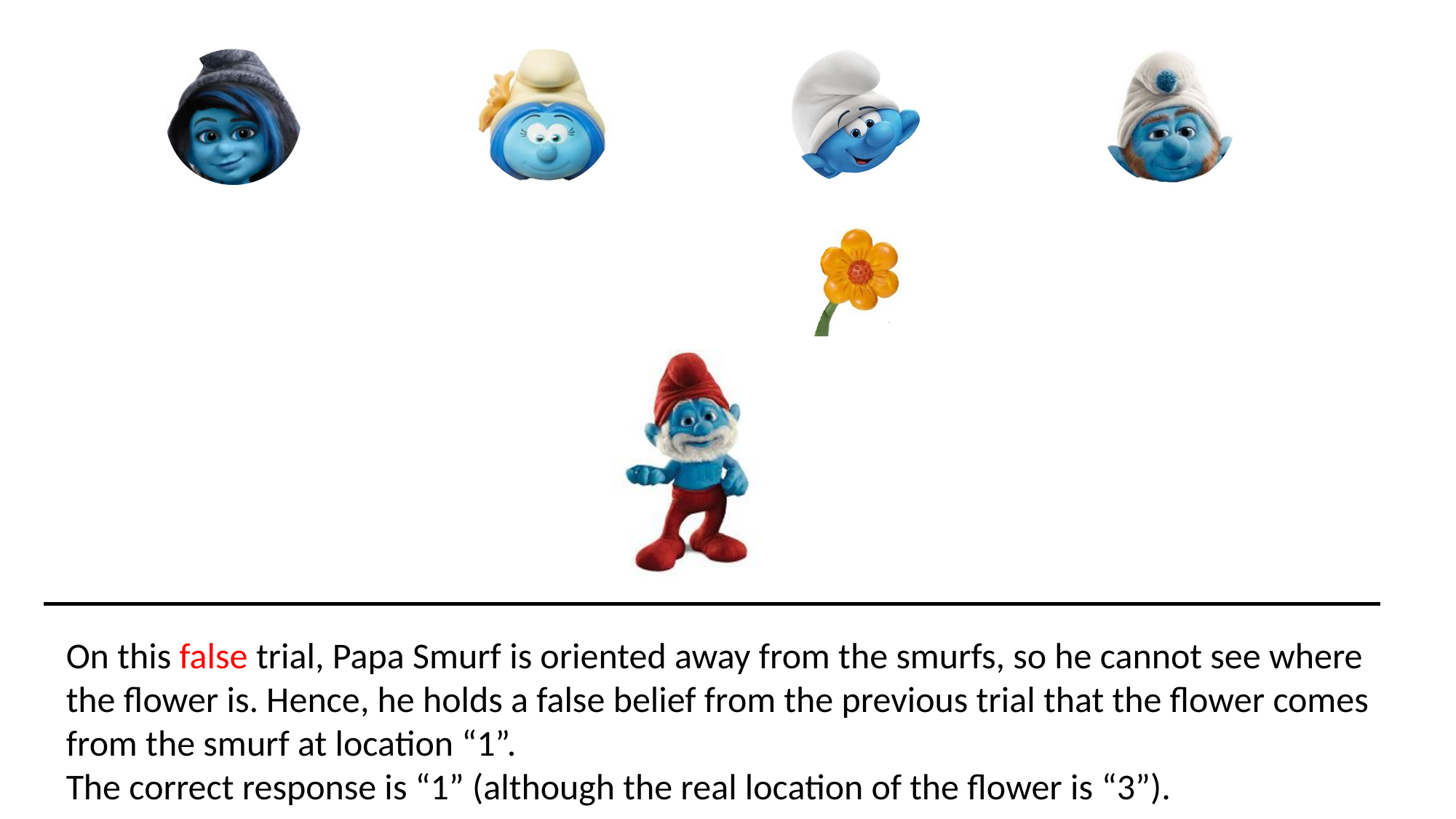

On this false trial, Papa Smurf is oriented away from the smurfs, so he cannot see where the flower is. Hence, he holds a false belief from the previous trial that the flower comes from the smurf at location “1”.
The correct response is “1” (although the real location of the flower is “3”).

## Slide 5
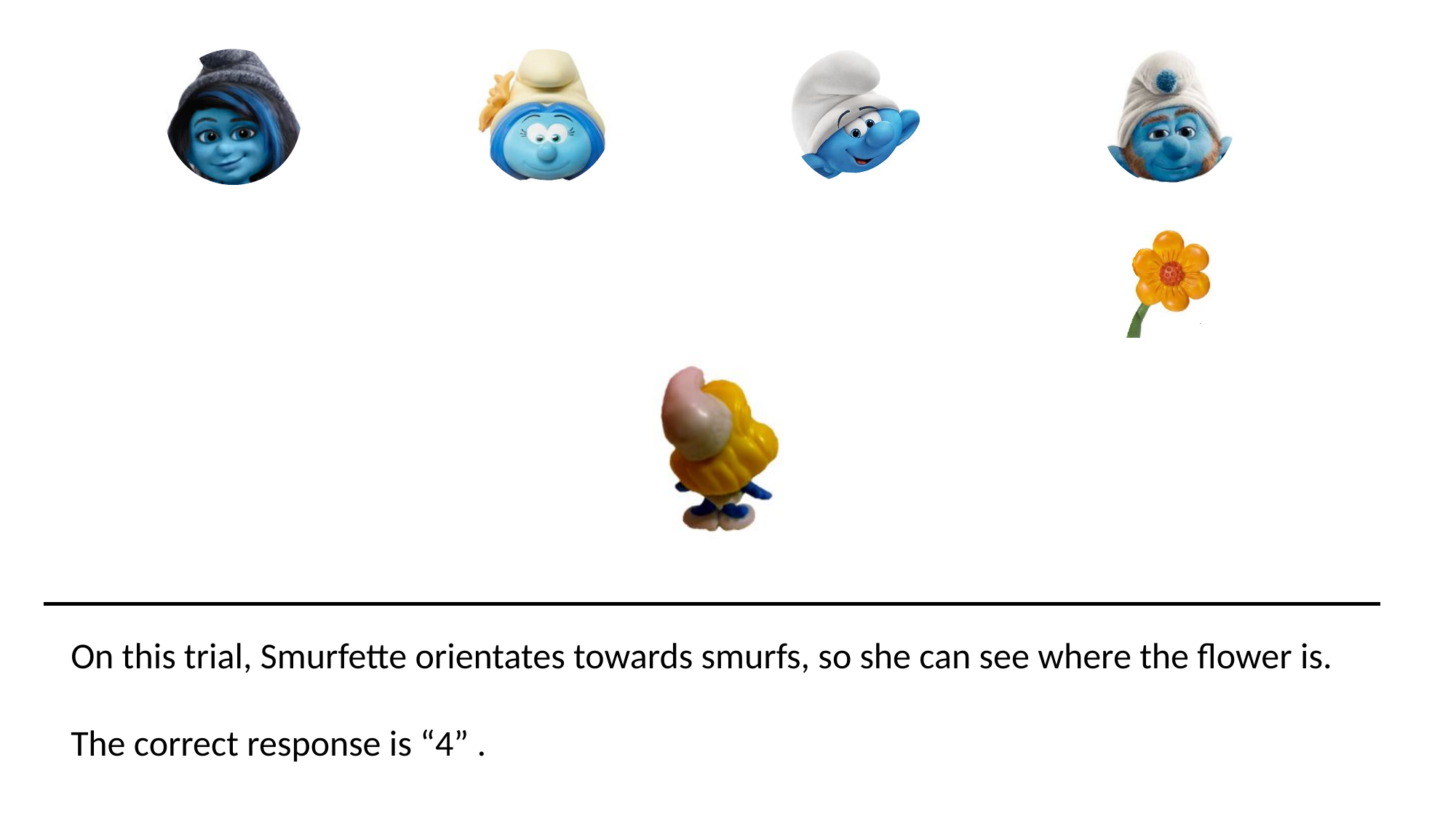

On this trial, Smurfette orientates towards smurfs, so she can see where the flower is.
The correct response is “4” .

## Slide 6
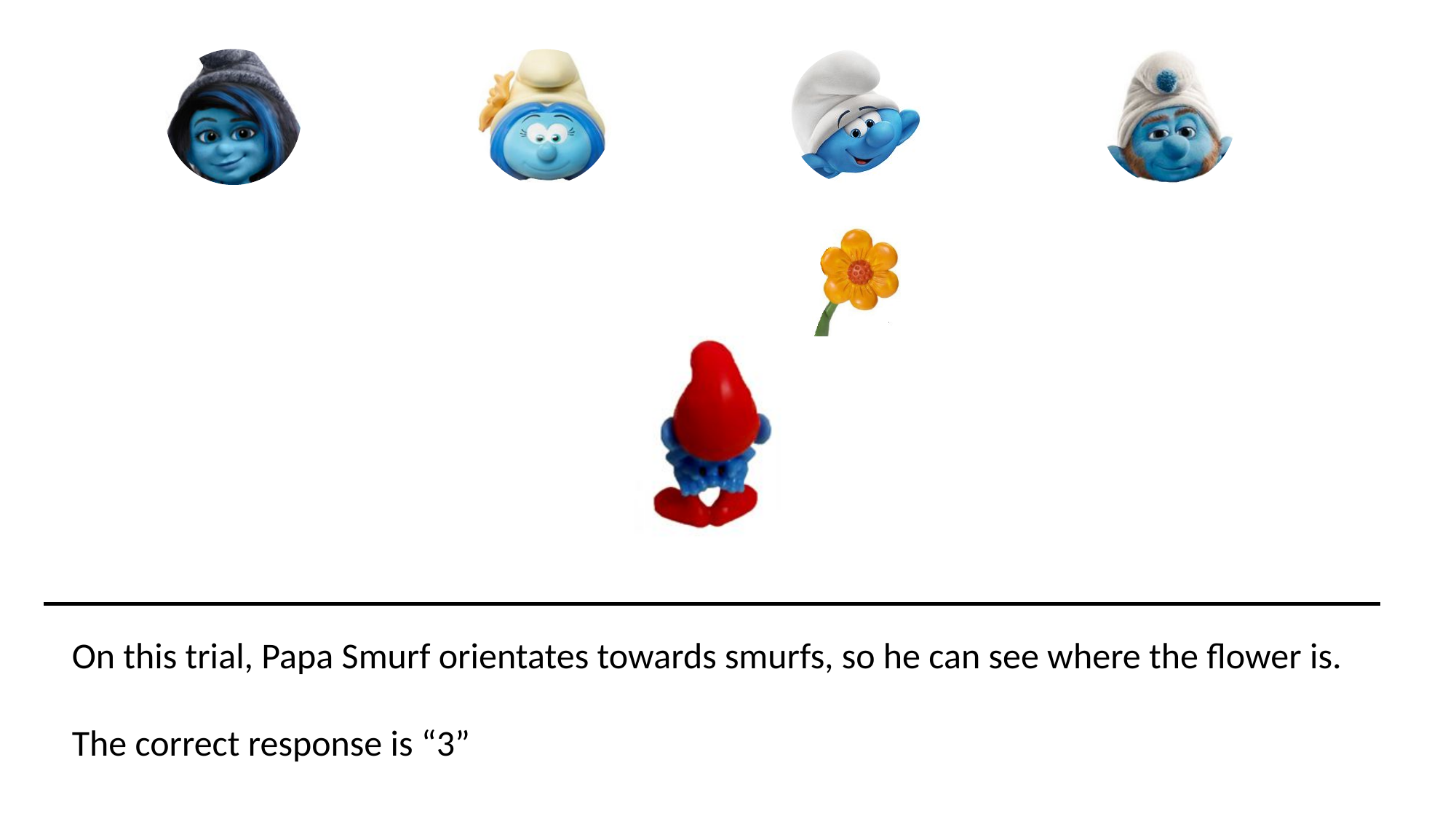

On this trial, Papa Smurf orientates towards smurfs, so he can see where the flower is.
The correct response is “3”

## Slide 7
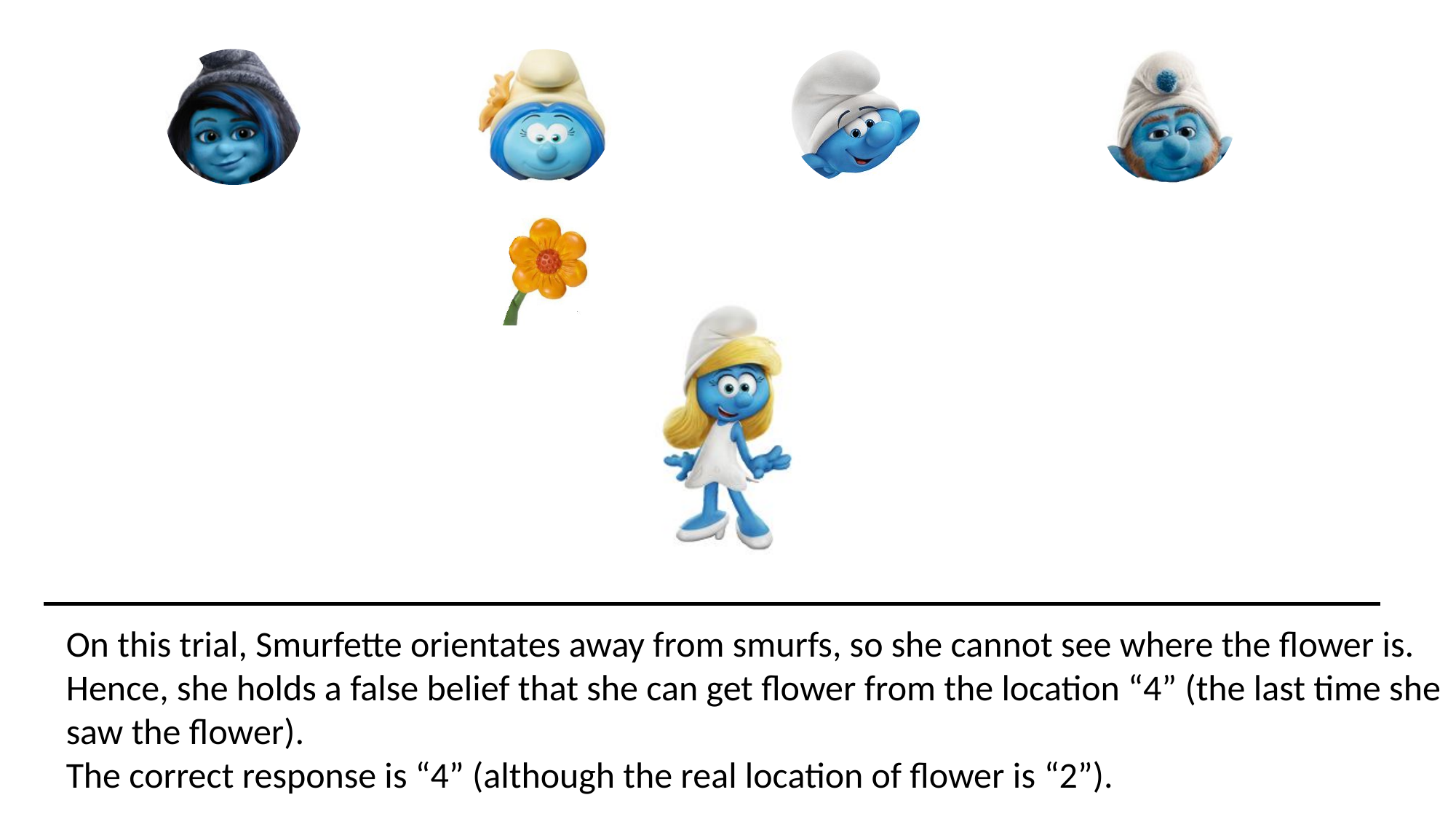

On this trial, Smurfette orientates away from smurfs, so she cannot see where the flower is.
Hence, she holds a false belief that she can get flower from the location “4” (the last time she saw the flower).
The correct response is “4” (although the real location of flower is “2”).

## Slide 8
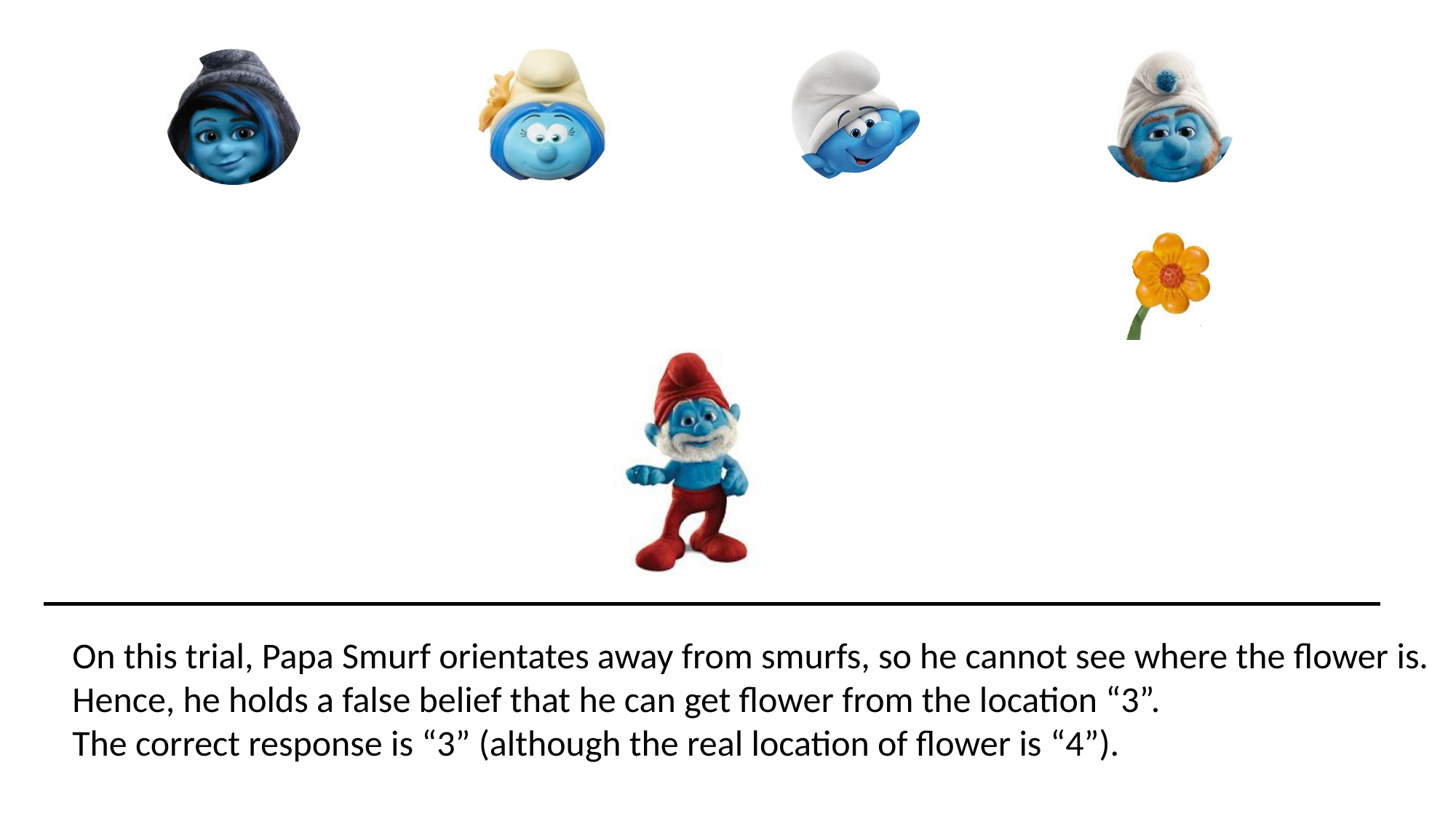

On this trial, Papa Smurf orientates away from smurfs, so he cannot see where the flower is.
Hence, he holds a false belief that he can get flower from the location “3”.
The correct response is “3” (although the real location of flower is “4”).

## Slide 9
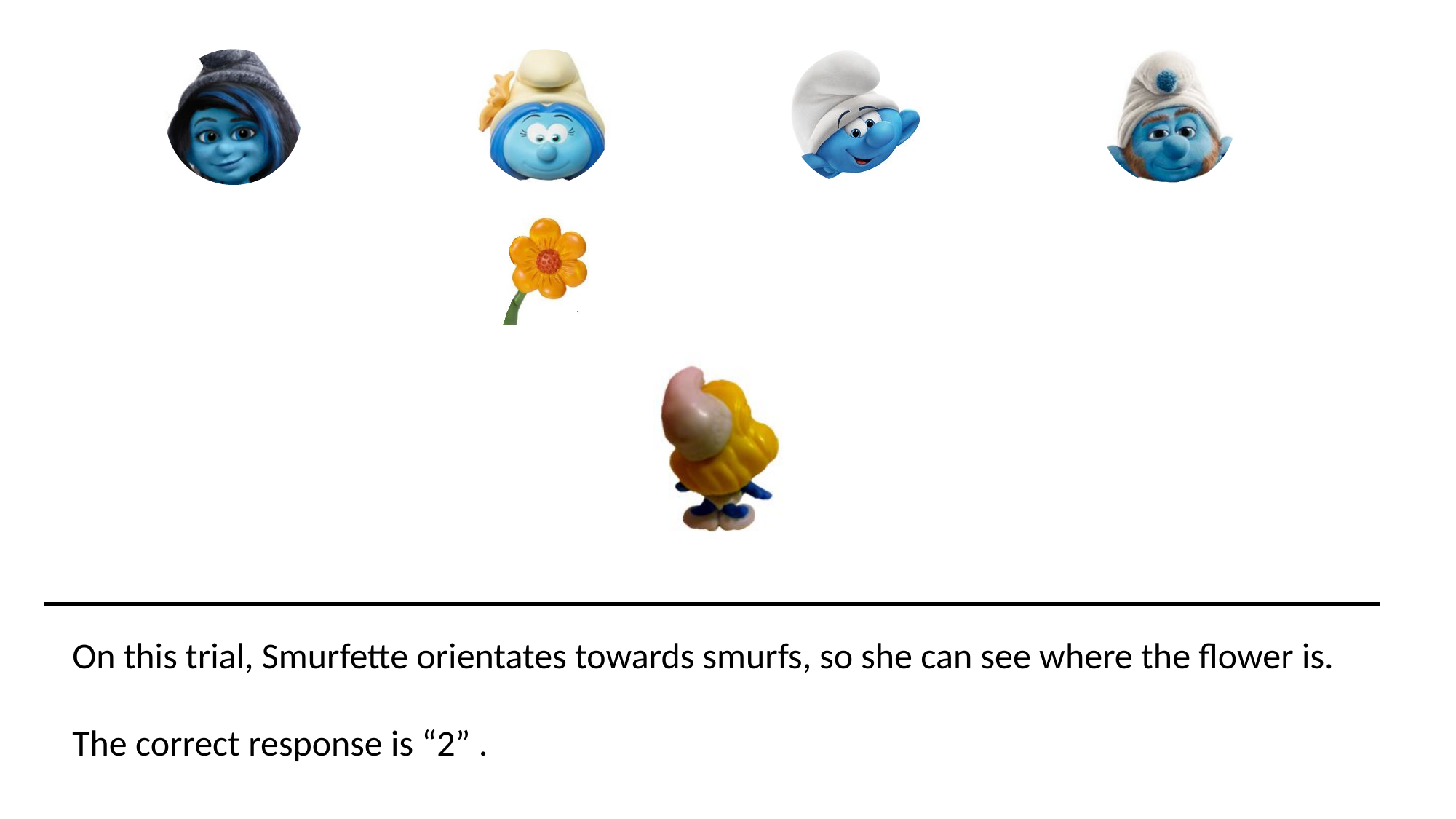

On this trial, Smurfette orientates towards smurfs, so she can see where the flower is.
The correct response is “2” .

## Slide 10
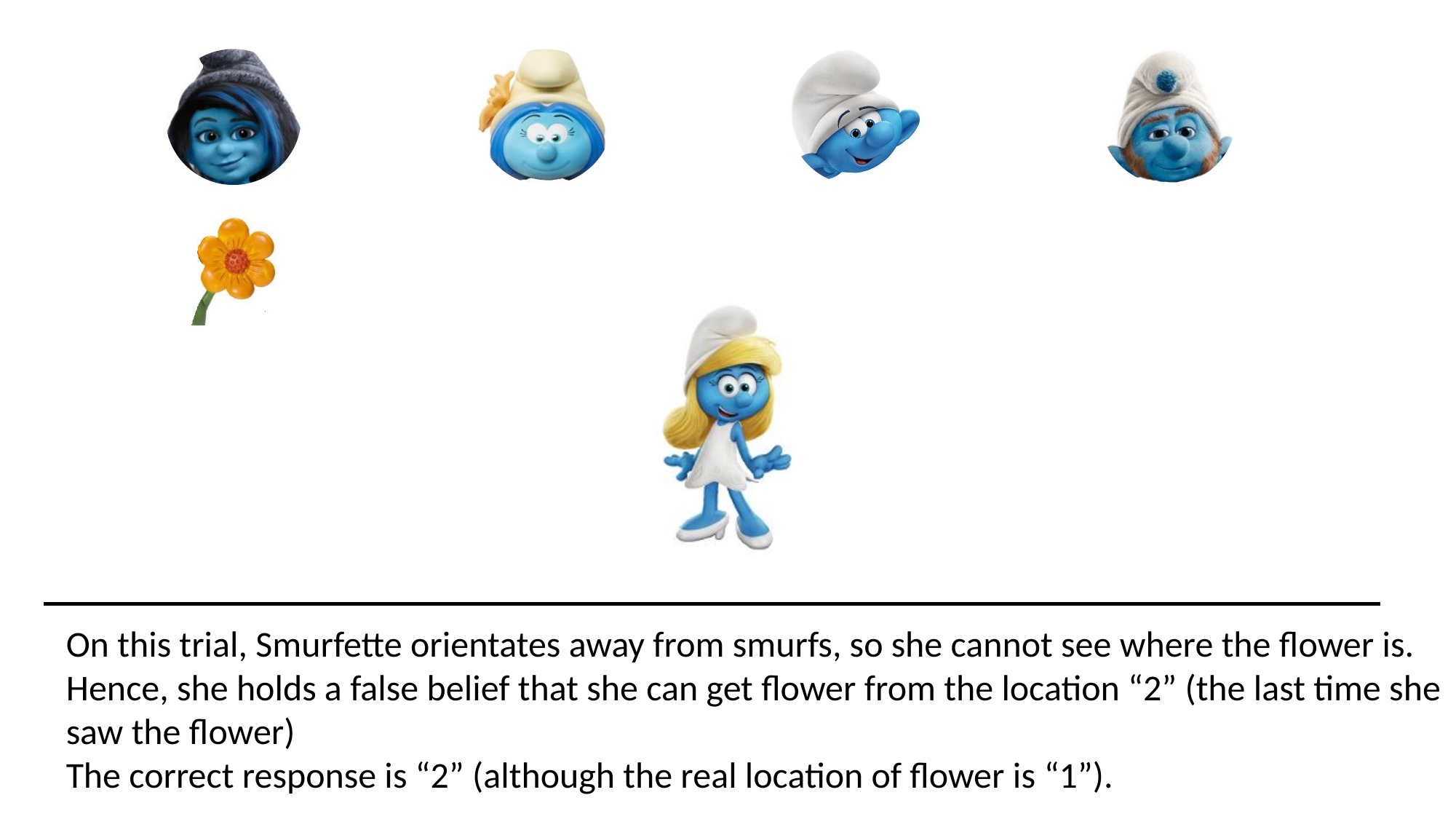

On this trial, Smurfette orientates away from smurfs, so she cannot see where the flower is.
Hence, she holds a false belief that she can get flower from the location “2” (the last time she saw the flower)
The correct response is “2” (although the real location of flower is “1”).
