# Supplementary material for: Implicit Learning of True and False Belief Sequences
Source: Front Psychol. 2021 Mar 26;12:643594. doi: 10.3389/fpsyg.2021.643594 (PMC8032999; doi:10.3389/fpsyg.2021.643594)

## Slide 1
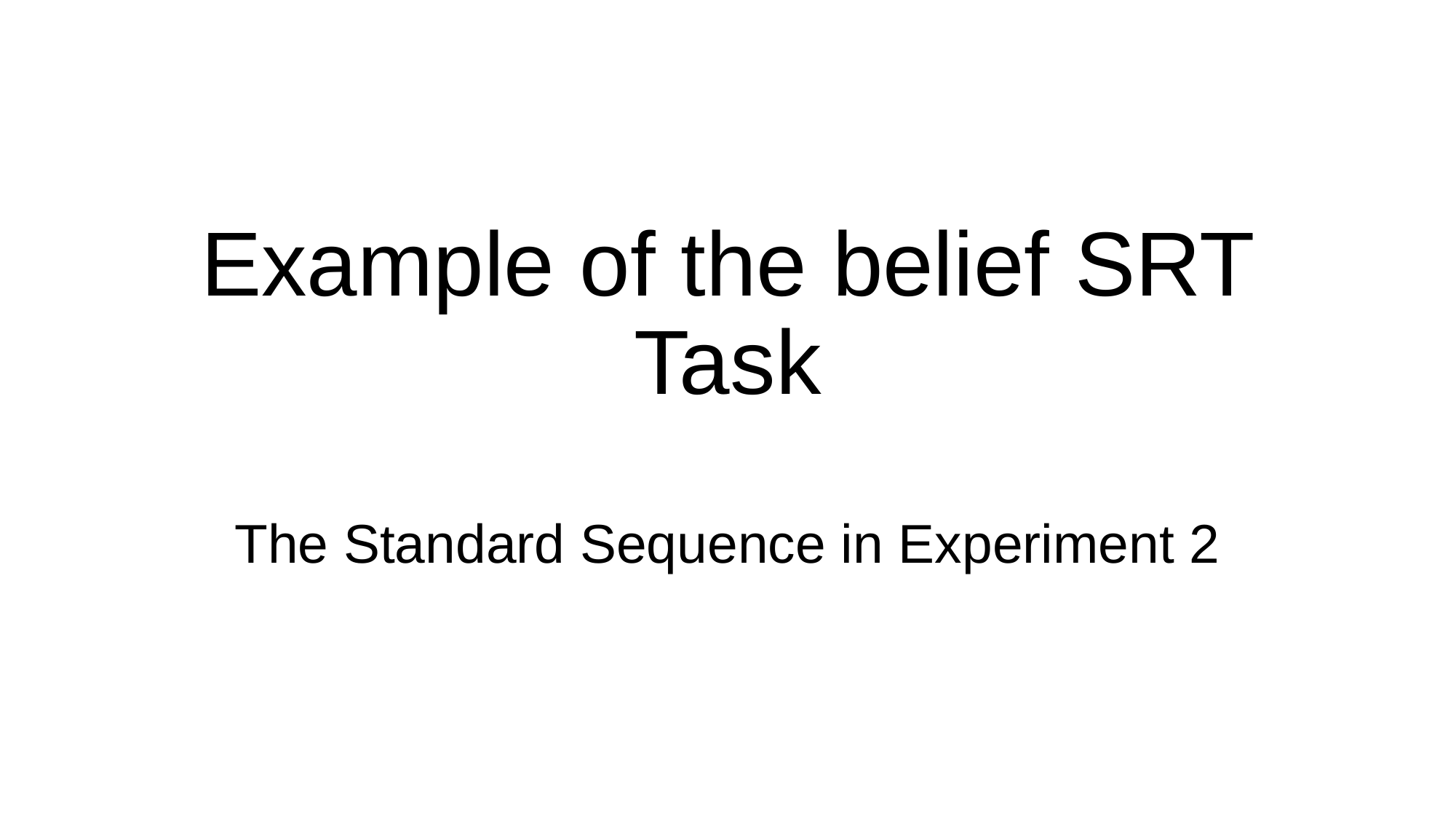

# Example of the belief SRT Task
The Standard Sequence in Experiment 2

## Slide 2
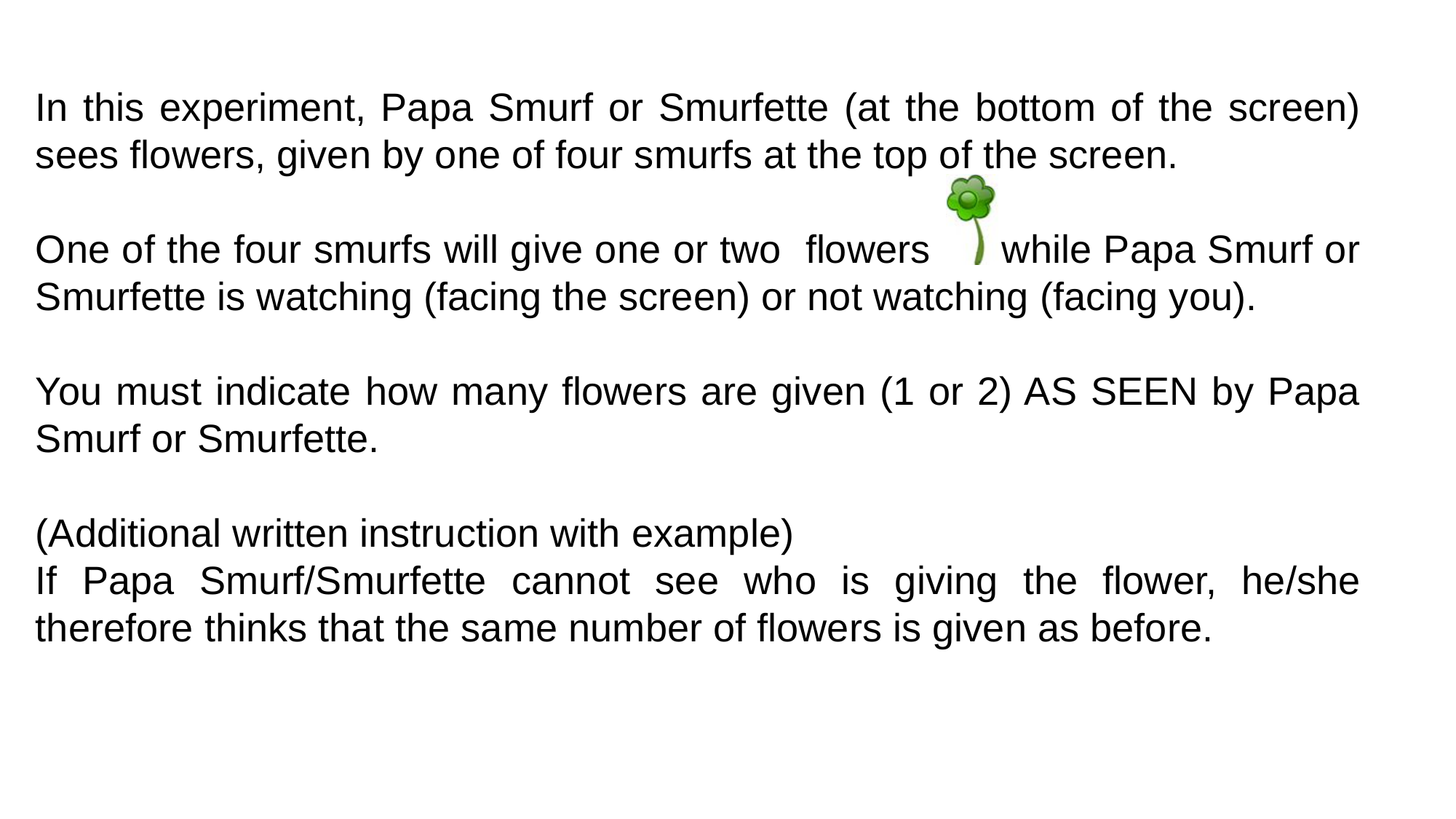

In this experiment, Papa Smurf or Smurfette (at the bottom of the screen) sees flowers, given by one of four smurfs at the top of the screen.
One of the four smurfs will give one or two flowers while Papa Smurf or Smurfette is watching (facing the screen) or not watching (facing you).
You must indicate how many flowers are given (1 or 2) AS SEEN by Papa Smurf or Smurfette.
(Additional written instruction with example)
If Papa Smurf/Smurfette cannot see who is giving the flower, he/she therefore thinks that the same number of flowers is given as before.

## Slide 3
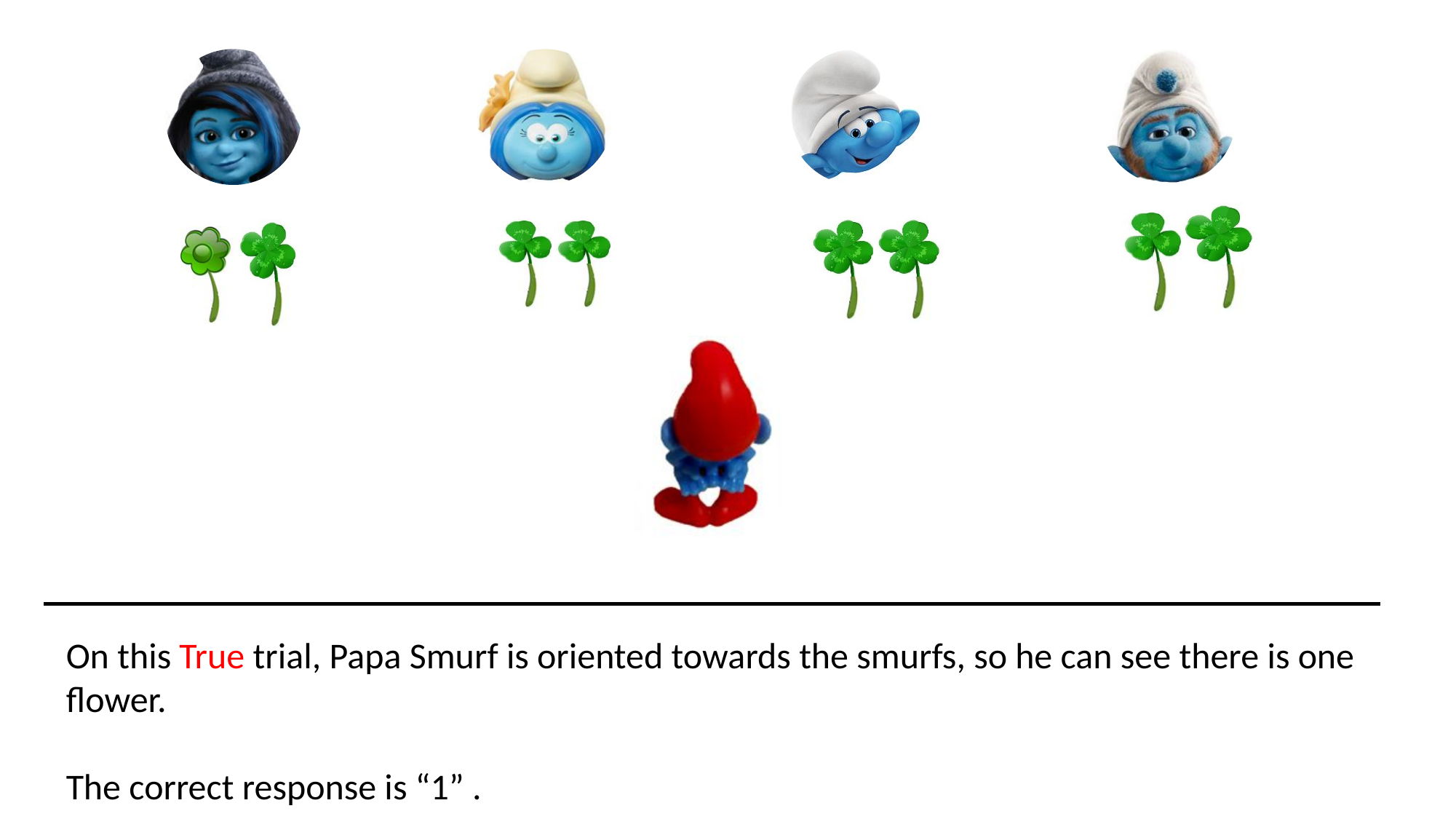

On this True trial, Papa Smurf is oriented towards the smurfs, so he can see there is one flower.
The correct response is “1” .

## Slide 4
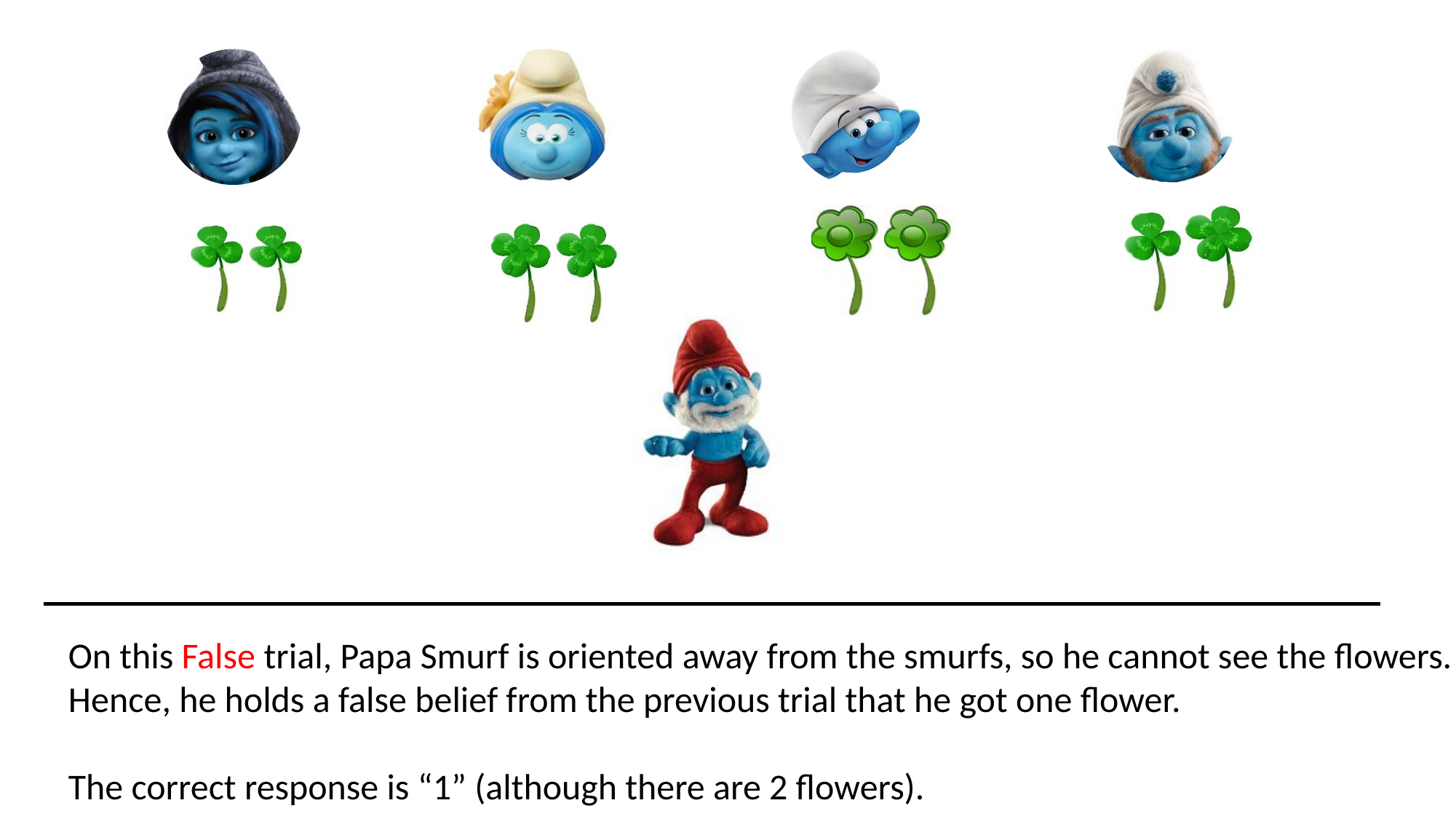

On this False trial, Papa Smurf is oriented away from the smurfs, so he cannot see the flowers.
Hence, he holds a false belief from the previous trial that he got one flower.
The correct response is “1” (although there are 2 flowers).

## Slide 5
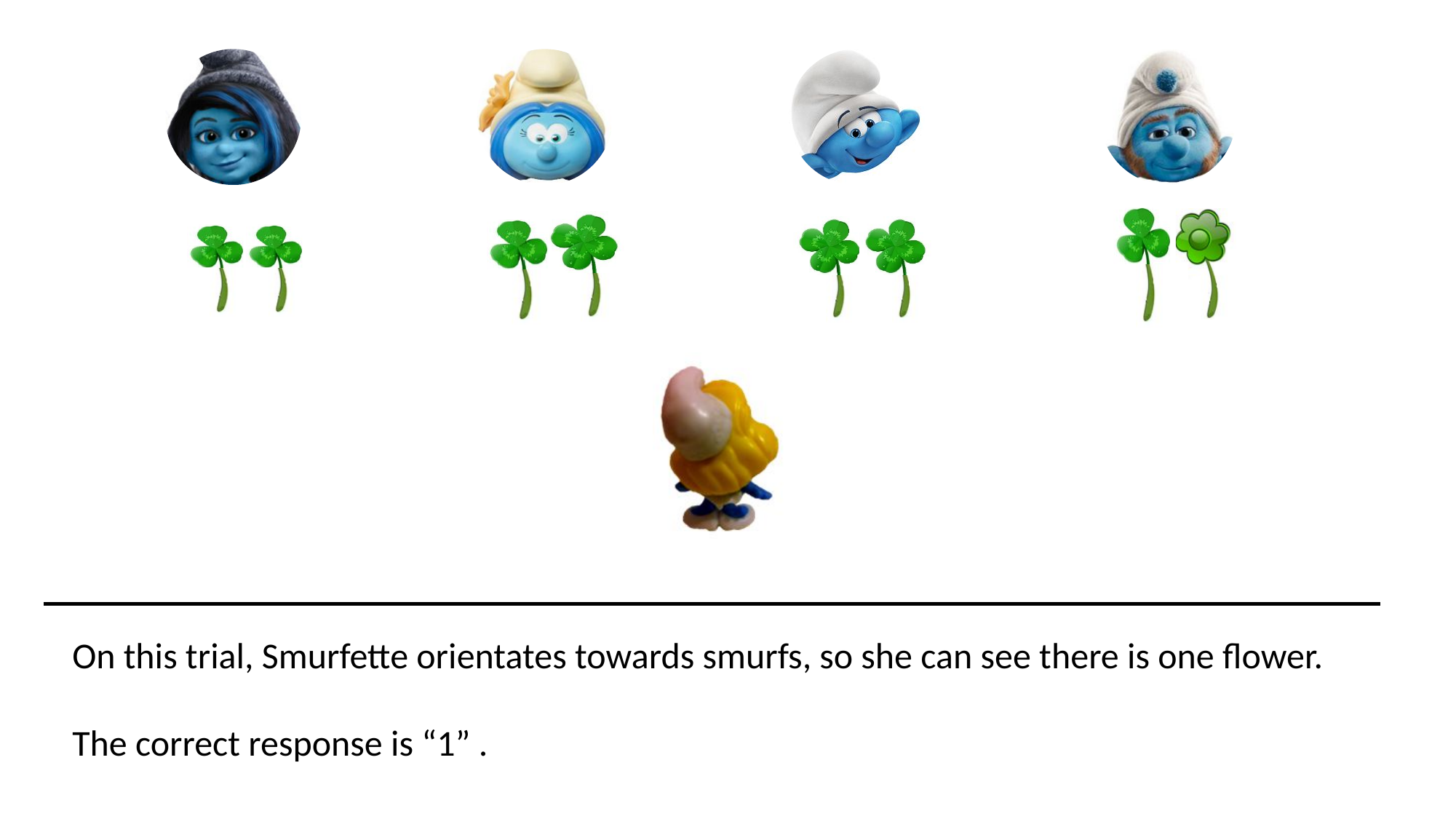

On this trial, Smurfette orientates towards smurfs, so she can see there is one flower.
The correct response is “1” .

## Slide 6
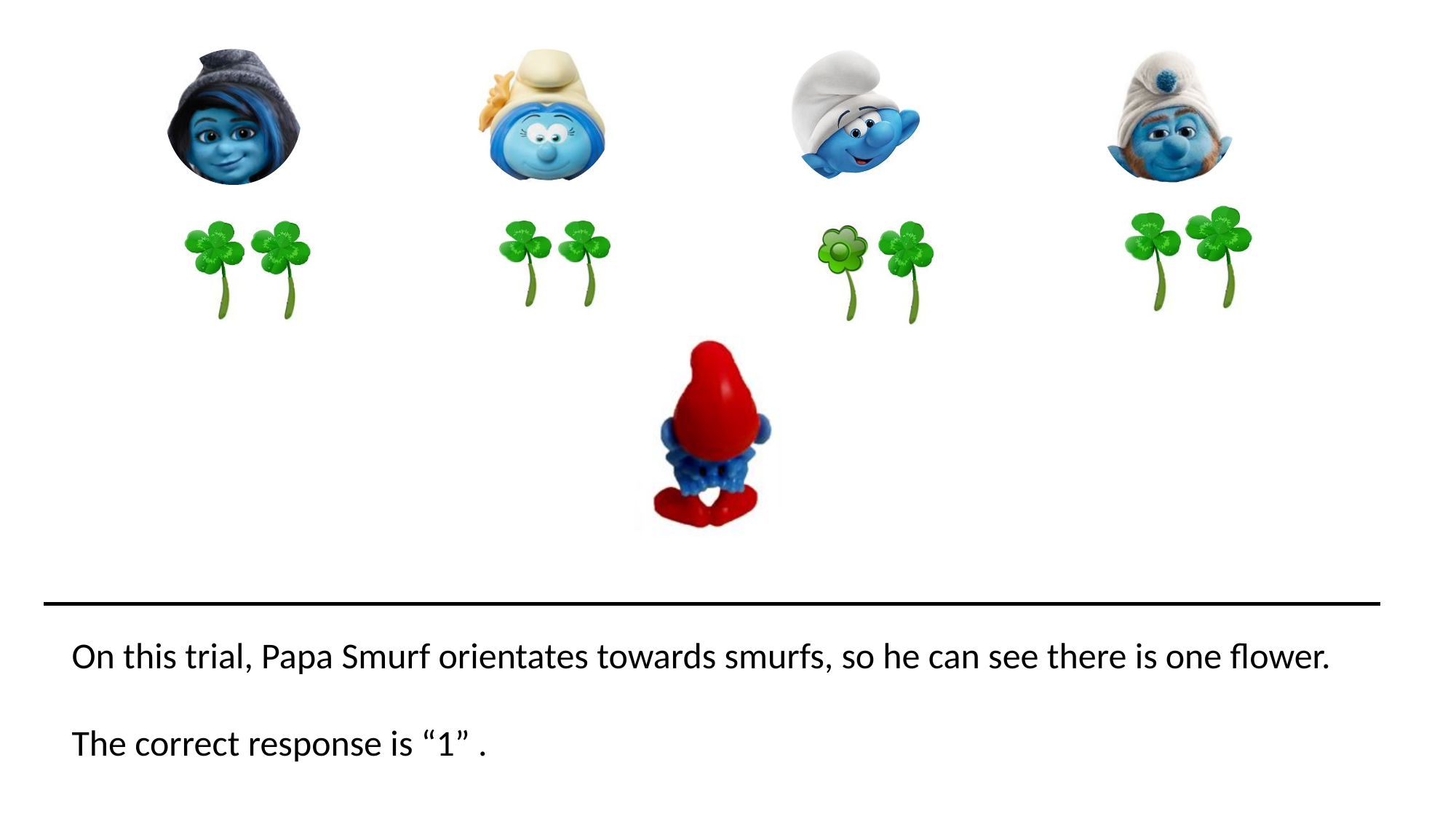

On this trial, Papa Smurf orientates towards smurfs, so he can see there is one flower.
The correct response is “1” .

## Slide 7
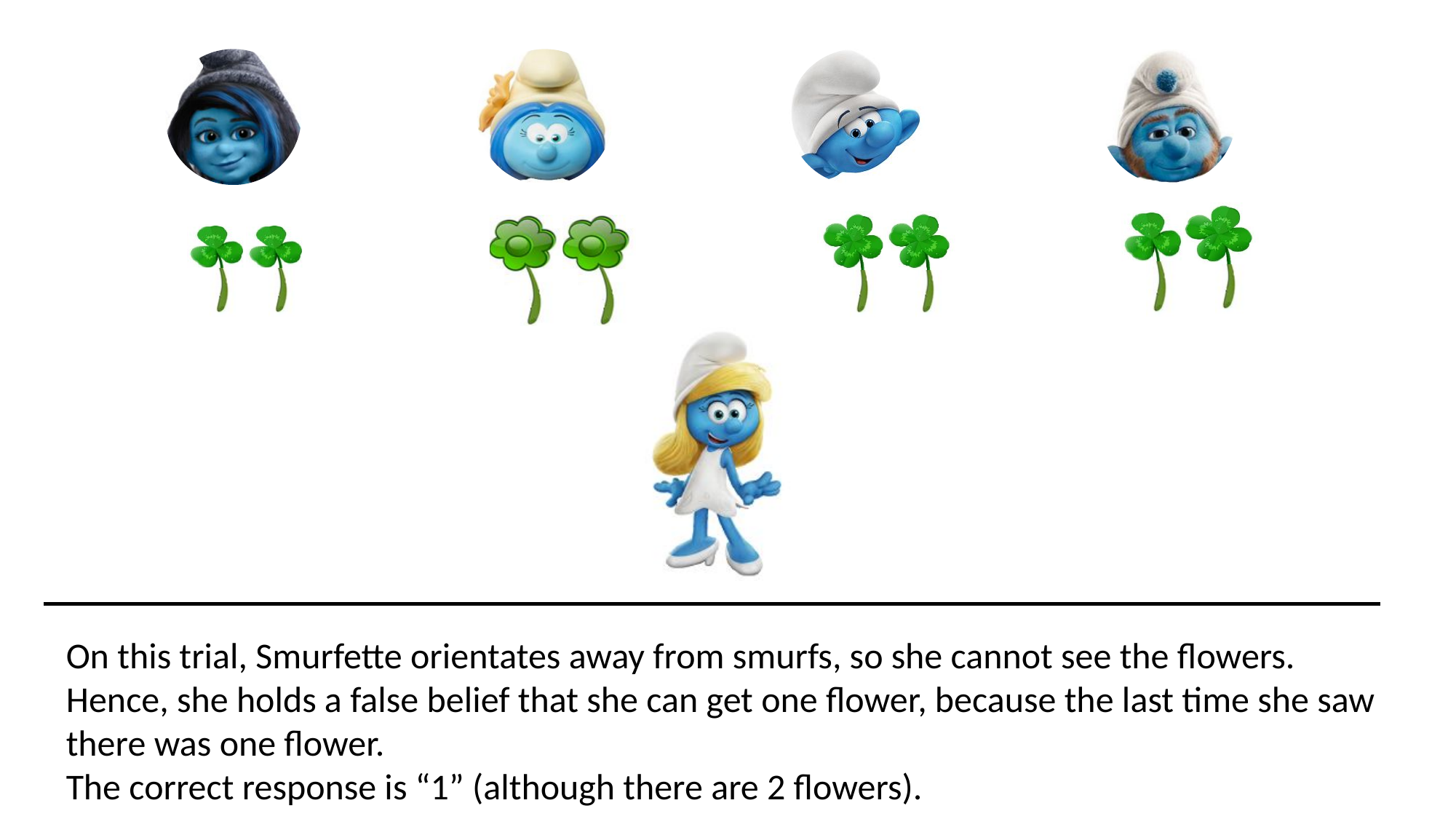

On this trial, Smurfette orientates away from smurfs, so she cannot see the flowers.
Hence, she holds a false belief that she can get one flower, because the last time she saw there was one flower.
The correct response is “1” (although there are 2 flowers).

## Slide 8
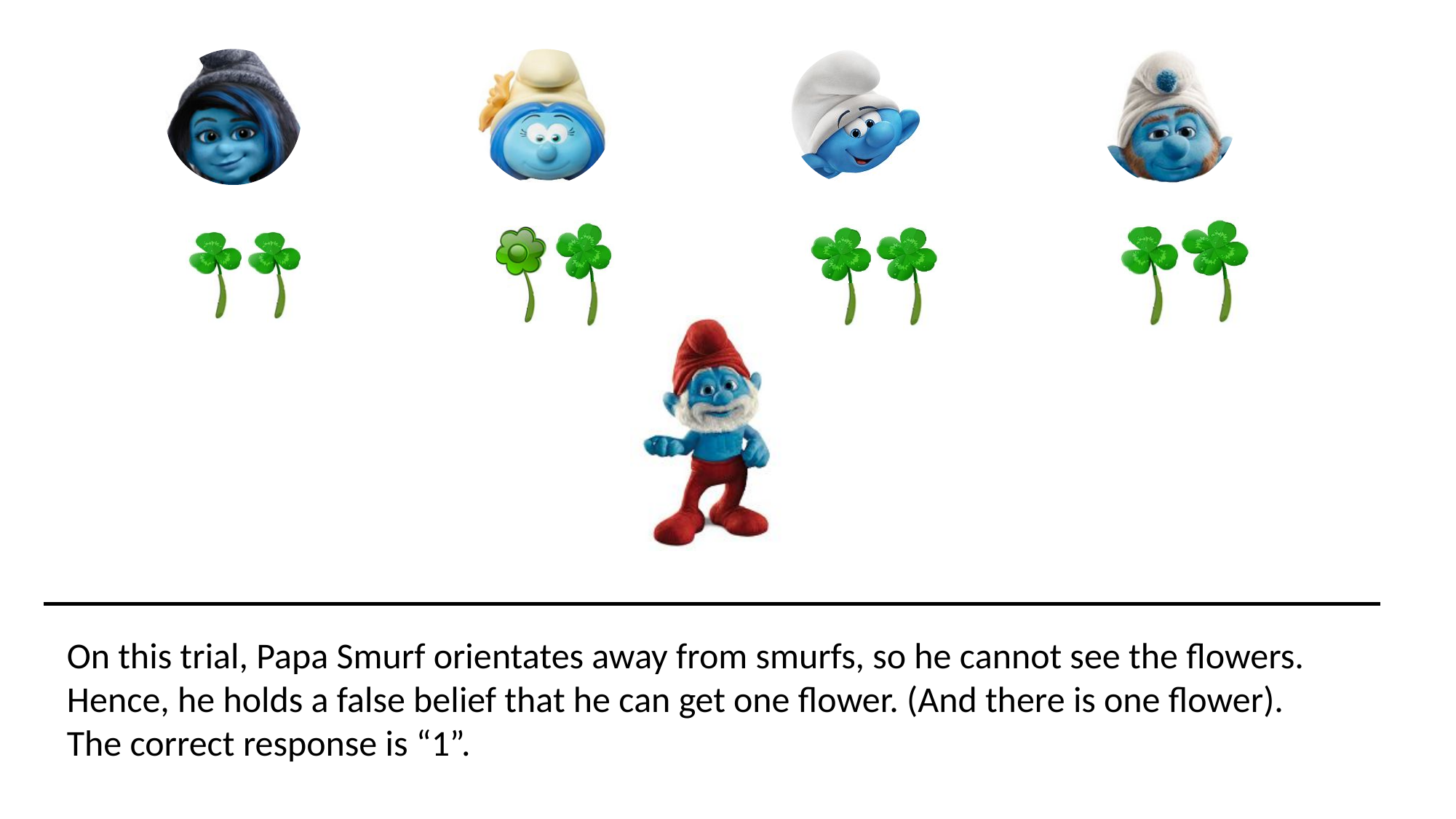

On this trial, Papa Smurf orientates away from smurfs, so he cannot see the flowers.
Hence, he holds a false belief that he can get one flower. (And there is one flower).
The correct response is “1”.

## Slide 9
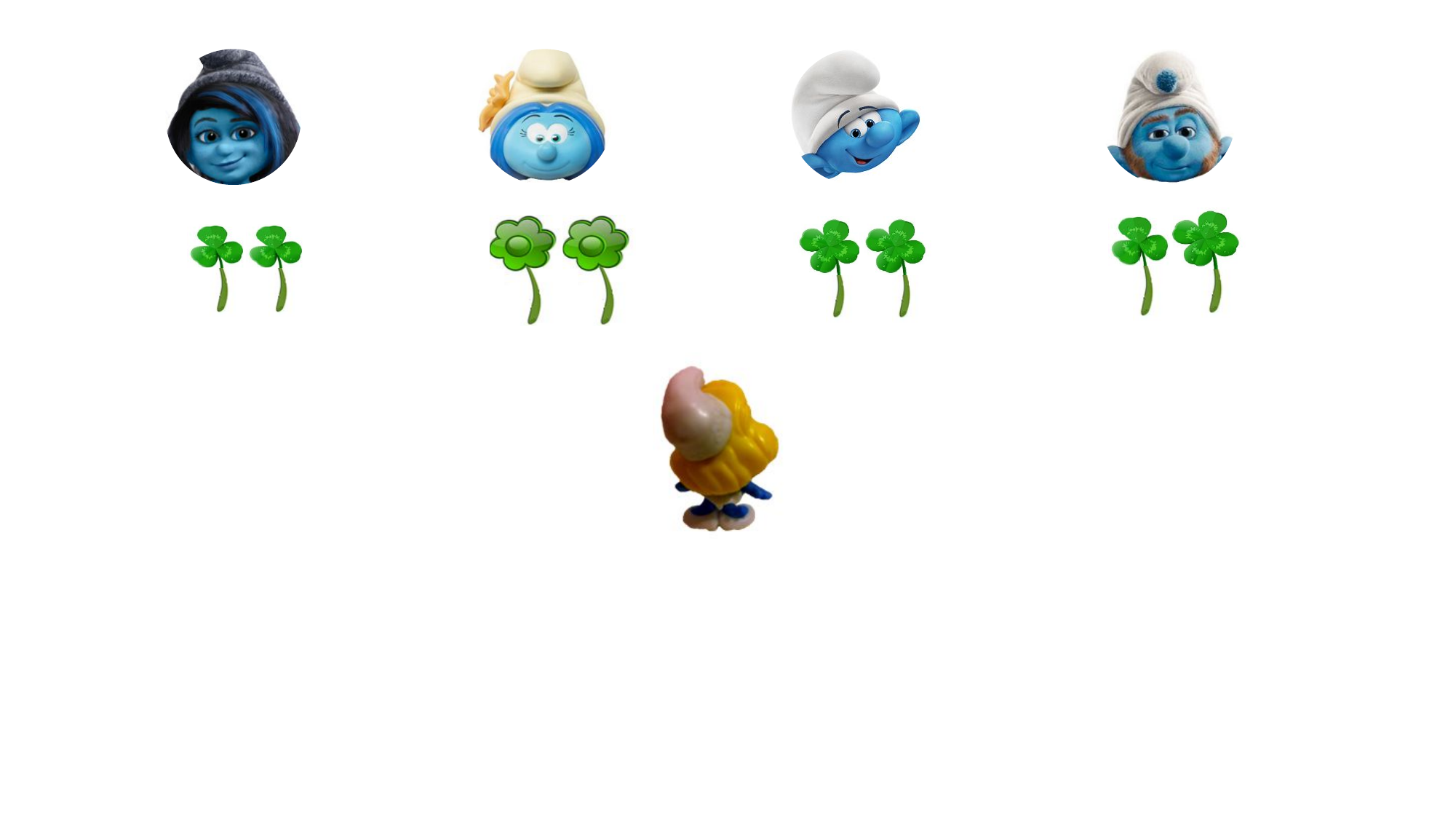

## Slide 10
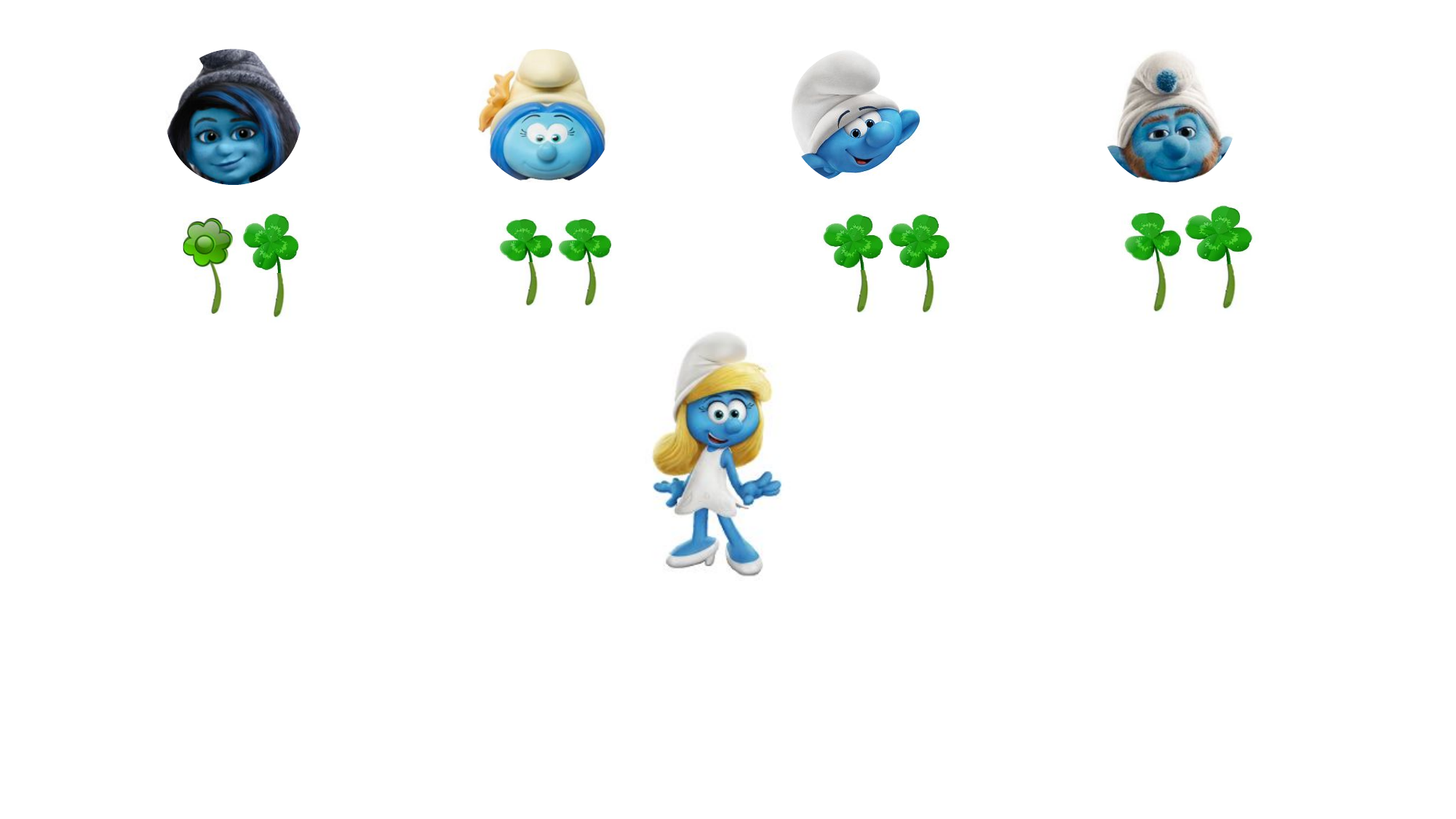

## Slide 11
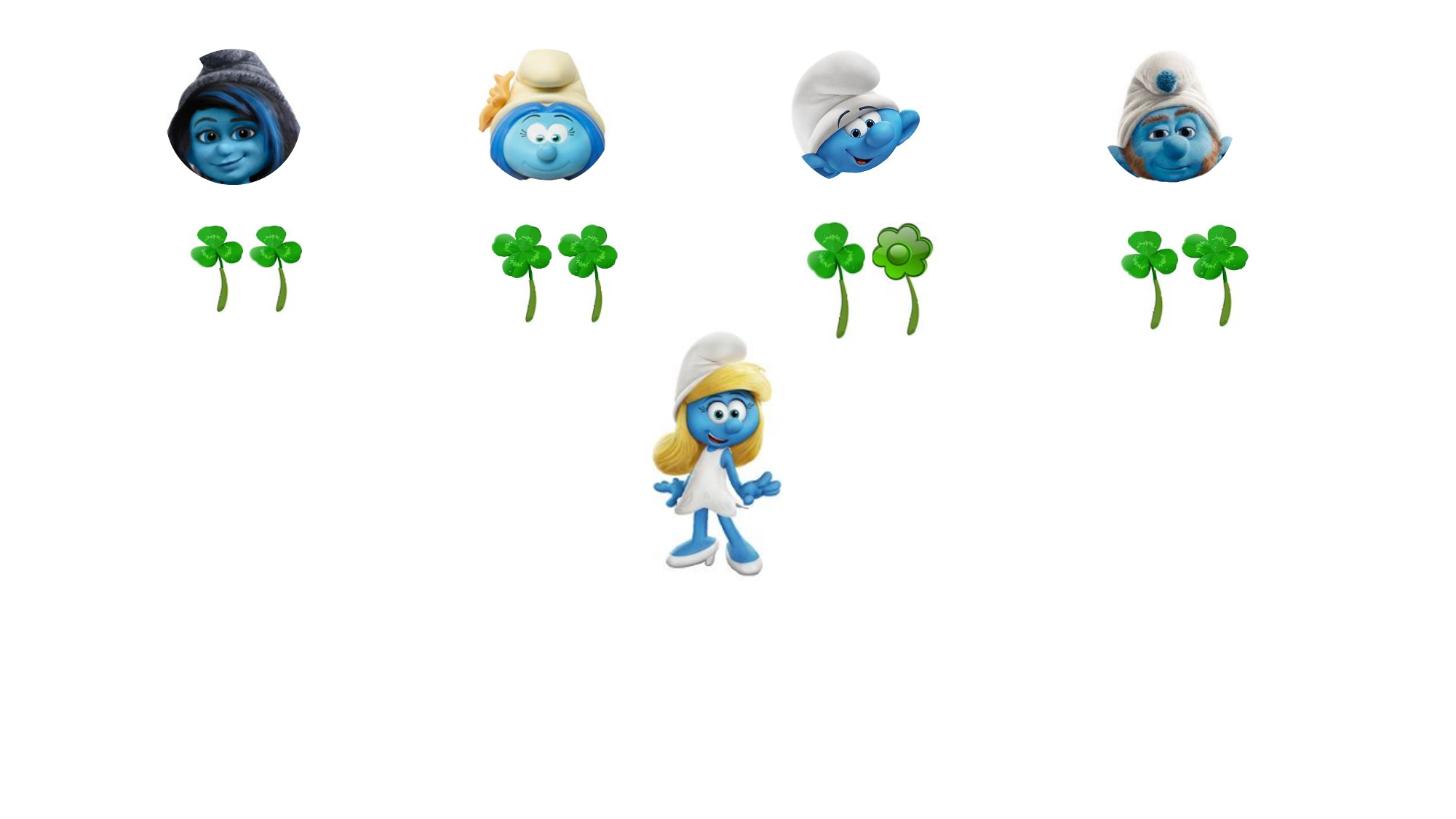

## Slide 12
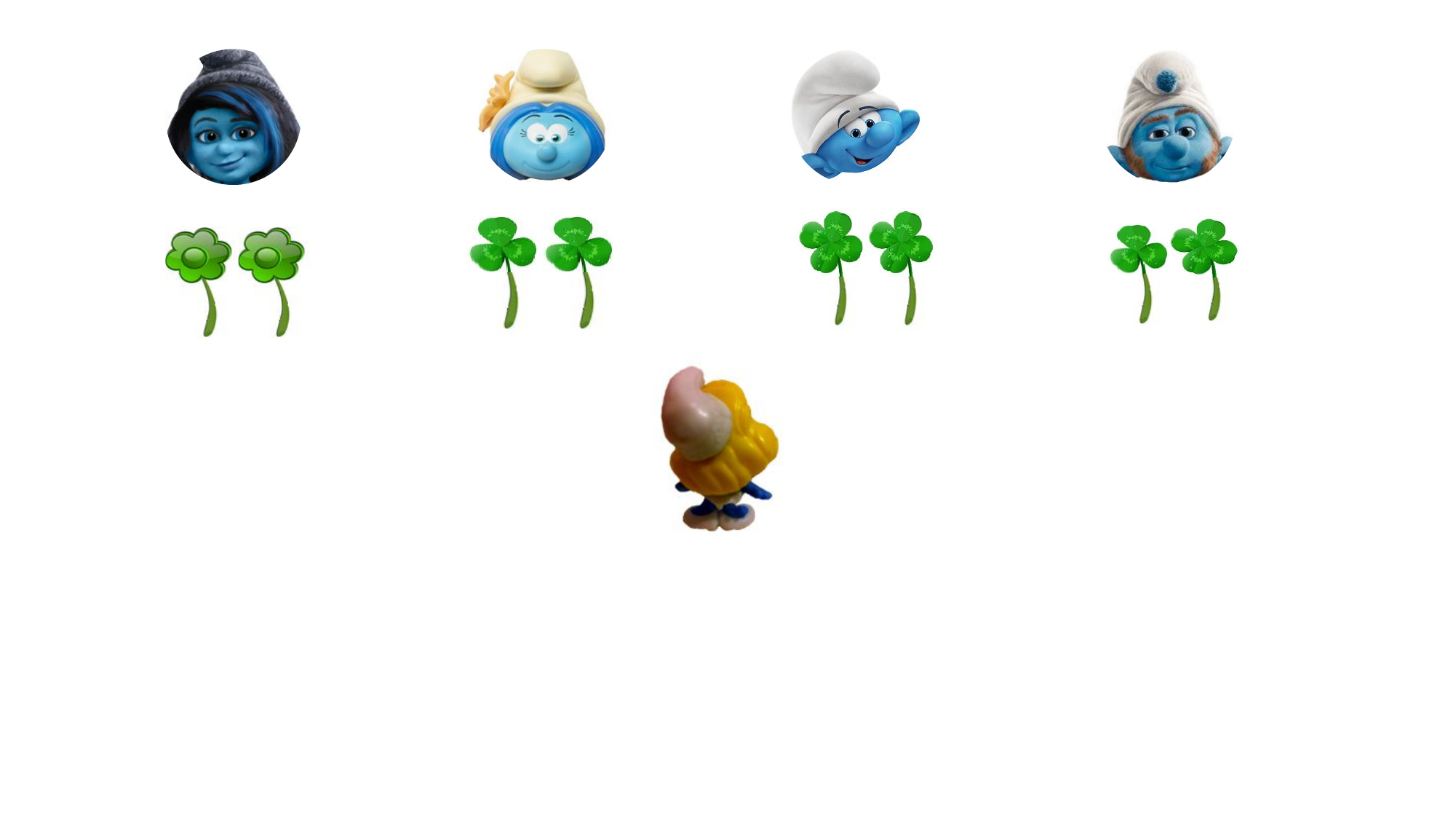

## Slide 13
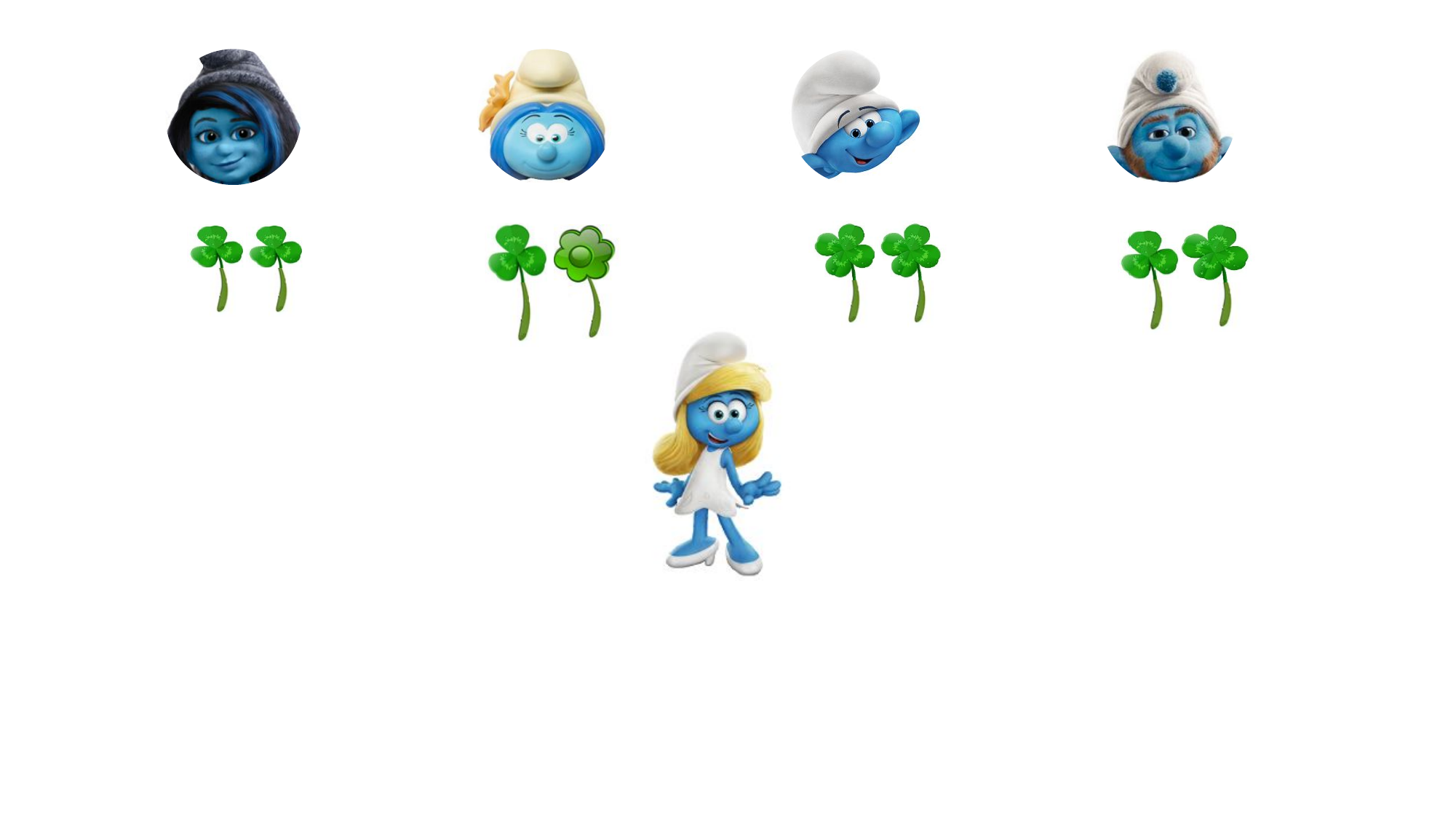

## Slide 14
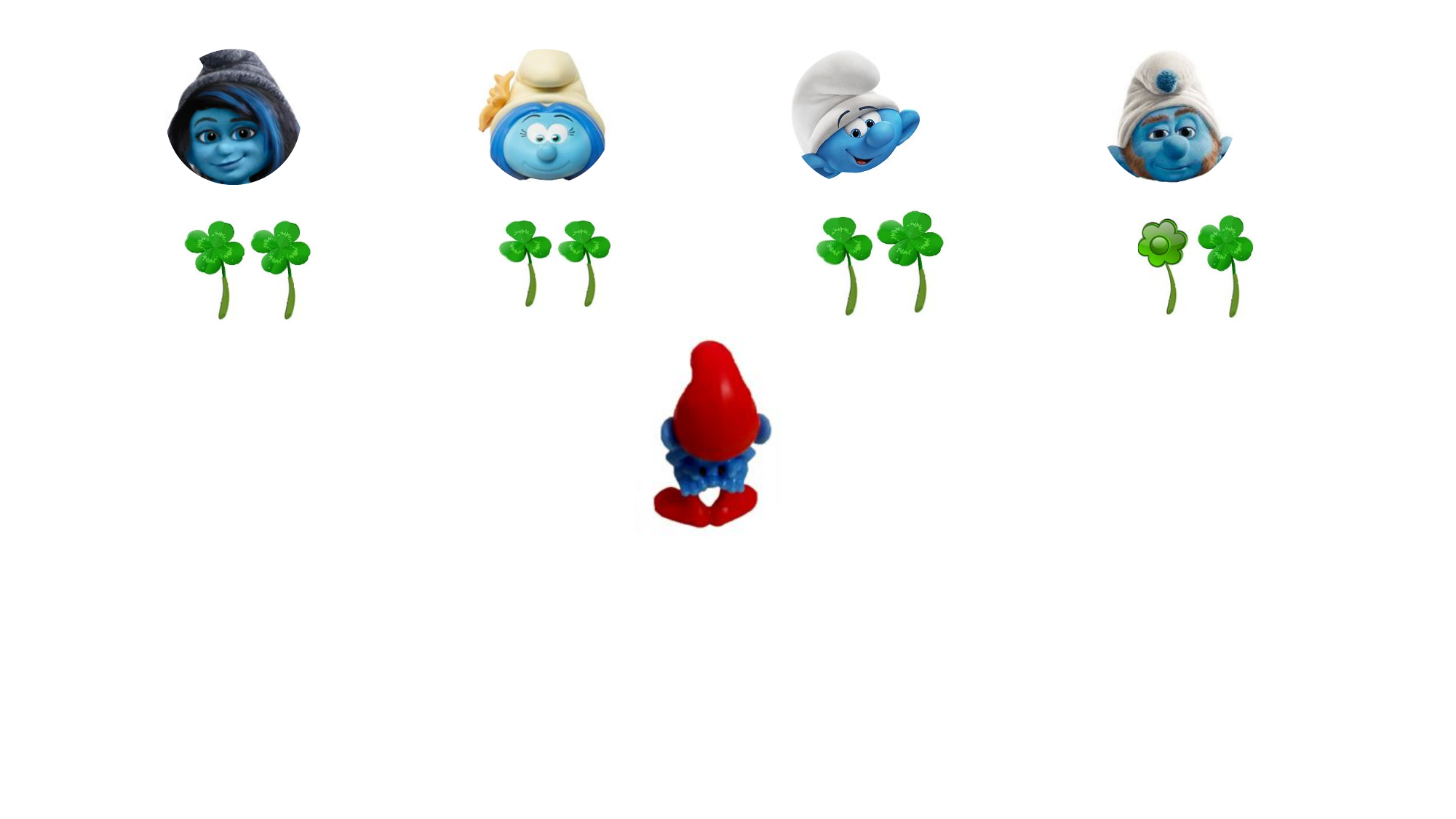

## Slide 15
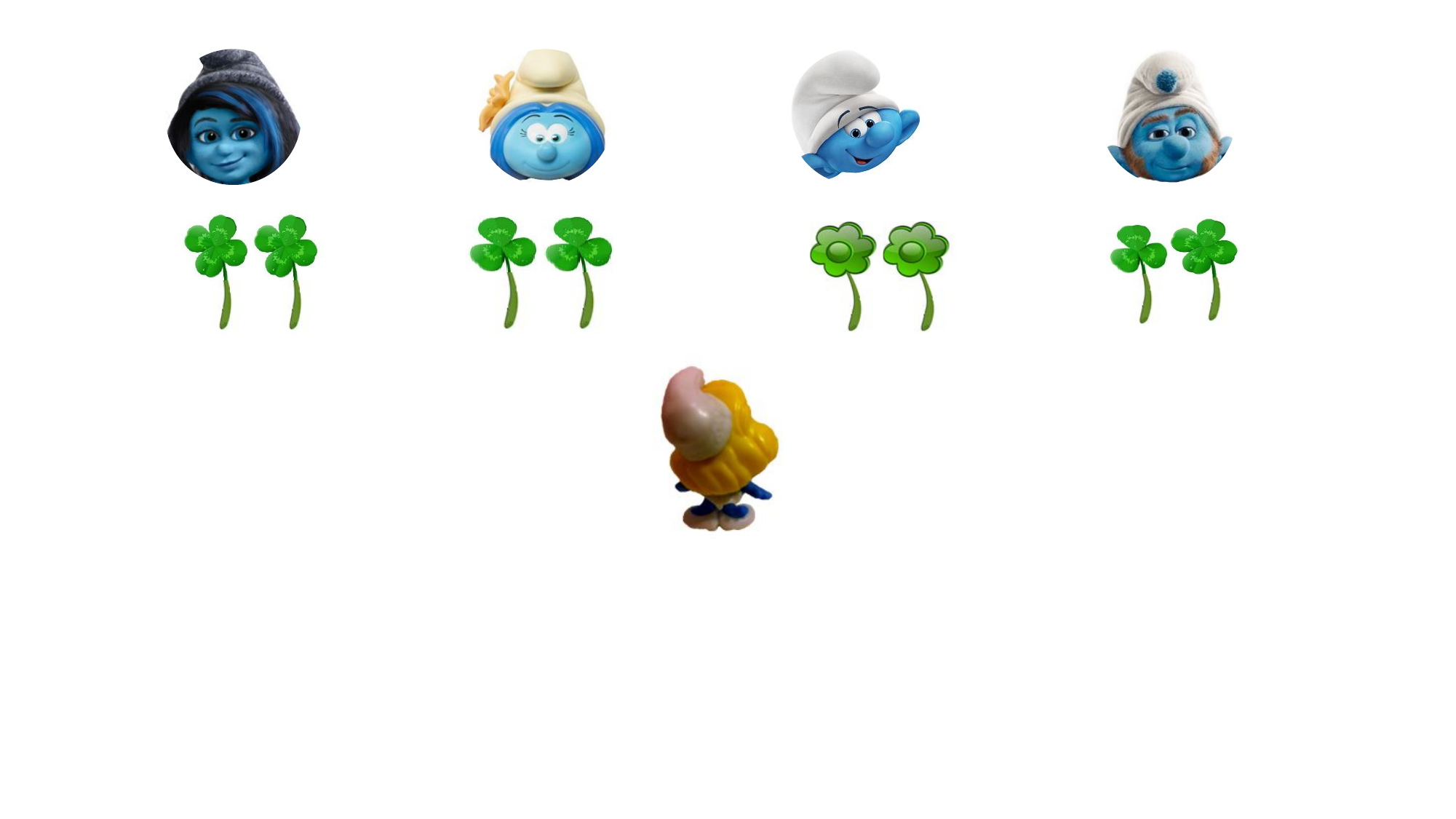

## Slide 16
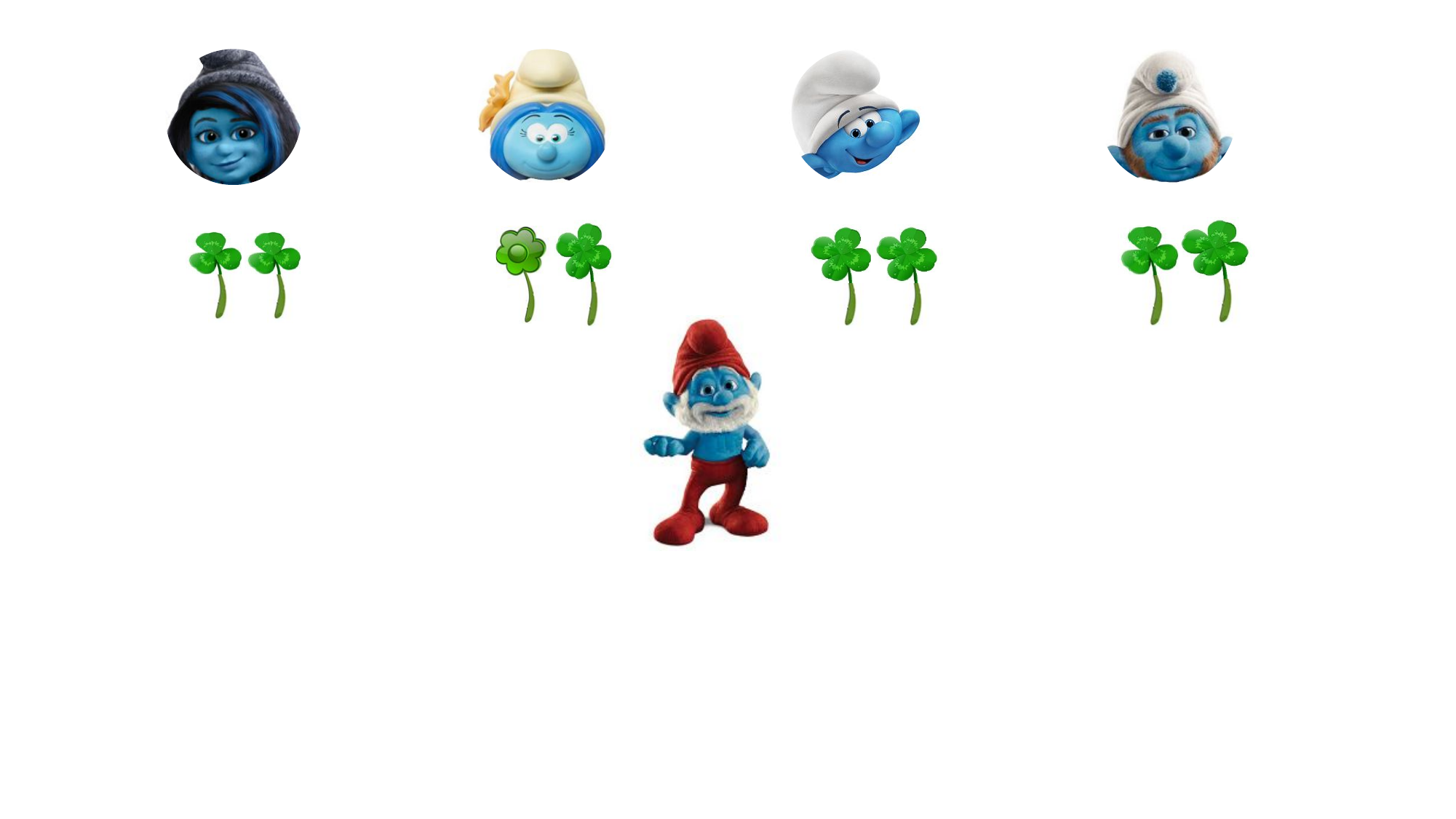

## Slide 17
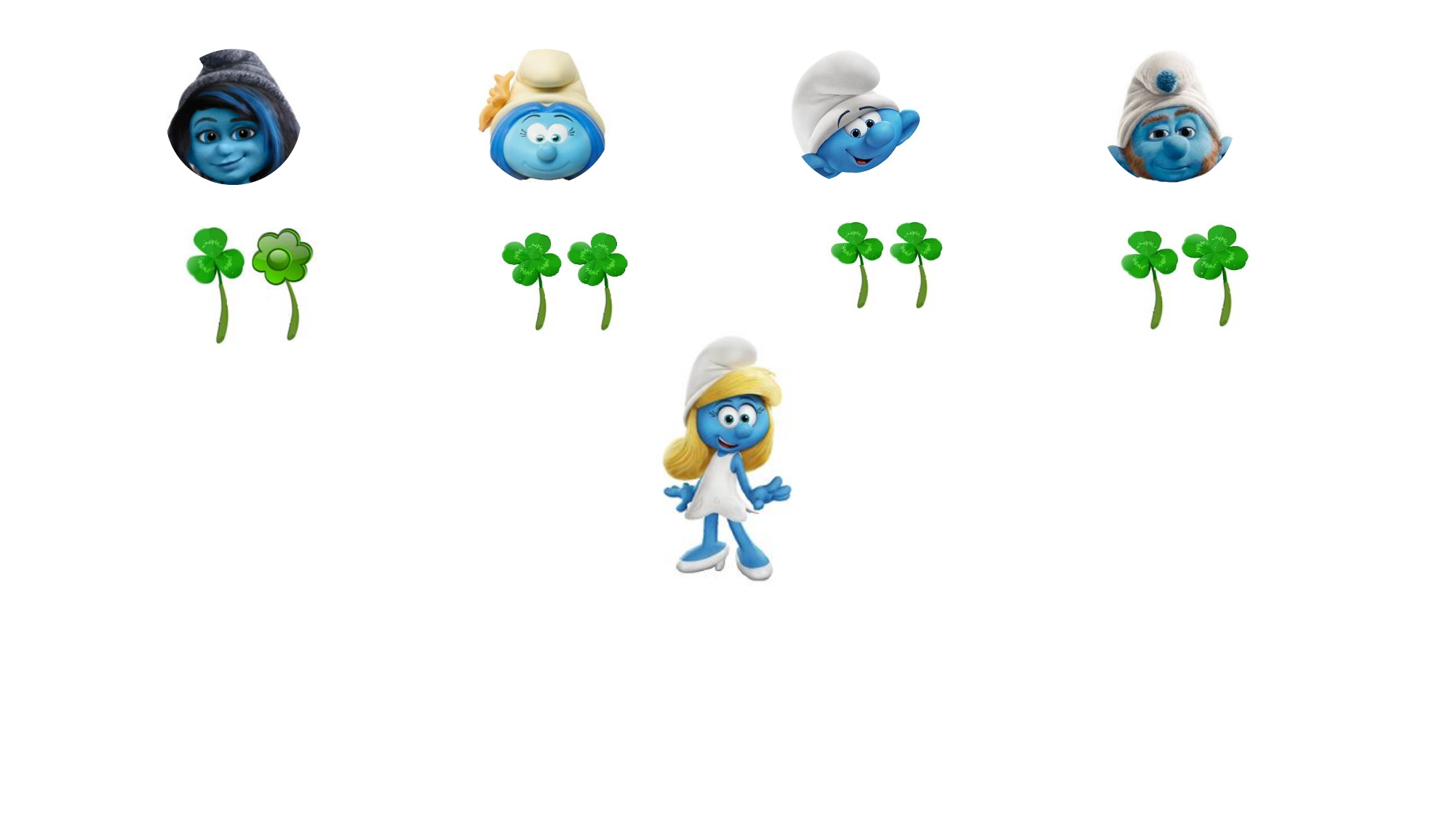

## Slide 18
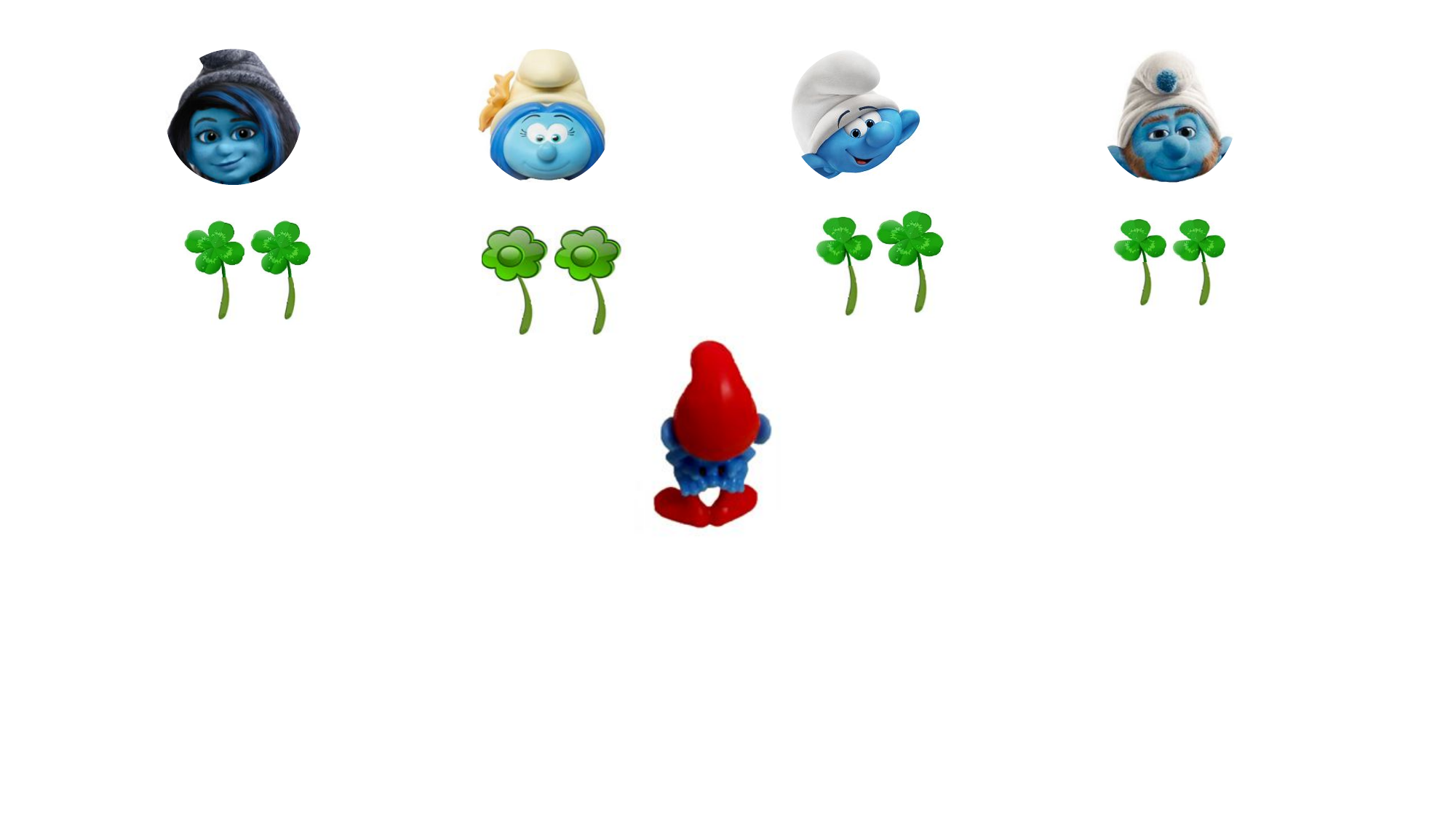

Supplement: Supplementary file 3 [file Presentation_2.PPTX]
